# Distinct placental molecular processes associated with early-onset and late-onset preeclampsia

**DOI:** 10.7150/thno.56141

**Published:** 2021-03-05

**Authors:** Zhonglu Ren, Yunfei Gao, Yue Gao, Guanmei Liang, Qian Chen, Sijia Jiang, Xiaoxue Yang, Cuixia Fan, Haizhen Wang, Jing Wang, Yi-Wu Shi, Chaoqun Xiao, Mei Zhong, Xinping Yang

**Affiliations:** 1 Center for genetics and Developmental Systems Biology, Department of Obstetrics and Gynecology, Nanfang Hospital, Southern Medical University, Guangzhou, 510515, China.; 2 Key Laboratory of Mental Health of the Ministry of Education, Guangdong-Hong Kong-Macao Greater Bay Area Center for Brain Science and Brain-Inspired Intelligence and Guangdong Key Laboratory of Psychiatric Disorders, School of Basic Medical Sciences, Southern Medical University, Guangzhou 510515, China.; 3 Department of Bioinformatics, School of Basic Medical Sciences, Southern Medical University, Guangzhou, 510515, China.; 4 College of Medical Information Engineering, Guangdong Pharmaceutical University, Guangzhou, 510006, China.; 5 Medicinal Information & Real-World Engineering Technology Center of Universities in Guangdong Province, Guangzhou, 510006, China.; 6 Institute of Neuroscience and Department of Neurology, The Second Affiliated Hospital of Guangzhou Medical University, Guangzhou, 510260, China.

**Keywords:** clinical subtypes, molecular mechanism, placenta, preeclampsia, transcriptome

## Abstract

**Background:** Patients with preeclampsia display a spectrum of onset time and severity of clinical presentation, yet the underlying molecular bases for the early-onset and late-onset clinical subtypes are not known. Although several transcriptome studies have been done on placentae from PE patients, only a small number of differentially expressed genes have been identified due to very small sample sizes and no distinguishing of clinical subtypes.

**Methods:** We carried out RNA-seq on 65 high-quality placenta samples, including 33 from 30 patients and 32 from 30 control subjects, to search for dysregulated genes and the molecular network and pathways they are involved in.

**Results:** We identified two functionally distinct sets of dysregulated genes in the two major subtypes: 2,977 differentially expressed genes in early-onset severe preeclampsia, which are enriched with metabolism-related pathways, notably transporter functions; and 375 differentially expressed genes in late-onset severe preeclampsia, which are enriched with immune-related pathways. We also identified some key transcription factors, which may drive the widespread gene dysregulation in both early-onset and late-onset patients.

**Conclusion:** These results suggest that early-onset and late-onset severe preeclampsia have different molecular mechanisms, whereas the late-onset mild preeclampsia may have no placenta-specific causal factors. A few regulators may be the key drivers of the dysregulated molecular pathways.

## Introduction

Preeclampsia (PE) is hypertension presenting after 20 weeks of gestation accompanied by one of the following features: proteinuria, maternal organ damage (including hepatic, renal and neurological), or hematological involvement such as thrombocytopenia, and/or uteroplacental dysfunction, such as fetal growth restriction and/or abnormal uteroplacental circulation 1, 2. The diagnostic criteria include systolic blood pressure higher than 140 mm Hg, and/or diastolic blood pressure above 90 mm Hg, in addition to proteinuria of over 300 mg/day 3. It can be classified as early- or late-onset with a gestational age cutoff of 34 weeks 2, 4-6. The global incidence rate is 4.6% of pregnancies 7, leading to 60,000 maternal deaths per year 8. The grave consequences of PE on maternal health are mainly attributed to cerebral edema, intracranial hemorrhage and eclampsia 9, which some studies have suggested to be caused by abnormal functions of the autonomic nervous system mediated by placenta-derived factors 10-12. PE has significant consequences on fetal development and growth, often leading to perinatal and infant morbidity or mortality and contributes preterm births 13 and ~15% cases of fetal growth restriction (FGR) 14, 15. In the long term, PE affects brain development and functions of the offspring 16, leading to intellectual disability 17, epilepsy 18, autism 19-21 and schizophrenia 22, 23.

Since the symptoms of a pregnant woman with PE usually resolve after delivery and histopathological placental changes can be detected at that time, the placenta has been perceived as the location of the root cause. A widely accepted disease model for the placental origin includes two stages of PE development 24, 25. During the first stage, abnormal placentation in early gestation plays a role, such as poor trophoblast invasion and incomplete vascular formation of spiral arteries, which lead to placenta dysfunctions; and in the second stage, the dysfunctional placenta releases factors into the maternal blood, which in turn lead to hypertension and organ damage 24, 25. This model promoted intensive studies on placentae as root cause of PE, and increasing knowledge leads to finer classification of stages 26. However, the molecular mechanisms originating in the placenta remain largely unknown, and there are insufficient molecular criteria for distinction of the clinical subtypes.

Some PE cases occur in families, suggesting genetic factors play a role in its pathogenesis. First degree relatives of women with PE have a 5-fold higher risk of developing the disease 27. Men born from a PE pregnancy are also at risk of fathering PE pregnancies 28, 29, demonstrating that the placental genotype contributes to PE. The heritability of PE is about 50% 30, 31. Genome-wide linkage studies on pedigrees have identified risk loci on chromosomes 2p13, 2q23, 11q23, 10q22, 22q12, 2p25, 9p13, 4q32 and 9p11 32-36. Genome-wide association studies (GWAS) have found some variants associated with PE 37-39.

Patients with early-onset PE have more severe maternal and perinatal complications than those with late-onset PE 4. We do not know, however, if early-onset PE and late-onset PE have a common or distinct underlying molecular pathogenesis. Numerous placental microarray studies failed to identify distinct molecular signatures, due to small sample sizes, improper grouping of clinical subtypes and insufficient analyzing the transcriptome data 40-47. Some analyses use no classification of samples 41, 46 and some focus on severe PE 47, or early-onset severe PE 48. Although several microarray studies contain both early- and late-onset PE placental samples, these studies investigated differential expression between all PE samples and controls, or between early and late PE 42, 44, 45, but did not investigate differential expression in subtypes by comparing them to normal controls. RNA-seq allows for full sequencing of transcriptomes including coding and noncoding transcripts, and therefore more informative for discovery of mechanisms and biomarkers 49. A recent RNA-seq study, done on placentae of 9 PE samples (in three pools) and 9 controls (in three pools), found 53 PE-associated genes 50. The pooling of the samples and absence of subtype classification greatly reduced the power for detecting dysregulated genes. Although these studies point to placental mechanisms underlying PE involved in angiogenesis 42, 45, immune functions 40 and hypoxia 47, it is still not clear whether the molecular pathways involved in the functional defects of placentae are different in clinical subtypes of this complex disorder. We speculate that, if transcriptomes are compared between more homogeneous samples of each particular subtype and normal subjects, more complete sets of dysregulated genes might be revealed for each subtype, which would provide basis for the study on molecular mechanisms and early diagnosis.

In order to systematically search for placental molecular signatures associated with clinical subtypes, we carried out transcriptome sequencing on 65 high quality placenta samples, including 33 from 30 PE patients and 32 from 30 normal subjects. Considering the placenta is a vascular structure of microvessels, we achieved high data quality by removing the blood contamination using a computational strategy. We obtained 2,977 differentially expressed genes for early-onset severe PE and 375 differentially expressed genes for late-onset severe PE. The two dysregulated gene sets in the two major clinical subtypes are associated with two distinct sets of pathways. Two distinct sets of transcription factors may be responsible for most of the gene dysregulation in the two major subtypes. These findings provide not only distinct molecular signatures for the two major clinical subtypes of PE, but also potential pathways and regulators for understanding the molecular mechanisms of this complex disease.

## Results

### Subtype classification based on clinical characteristics and sample clustering based on RNA-seq data

**Figure 1:**
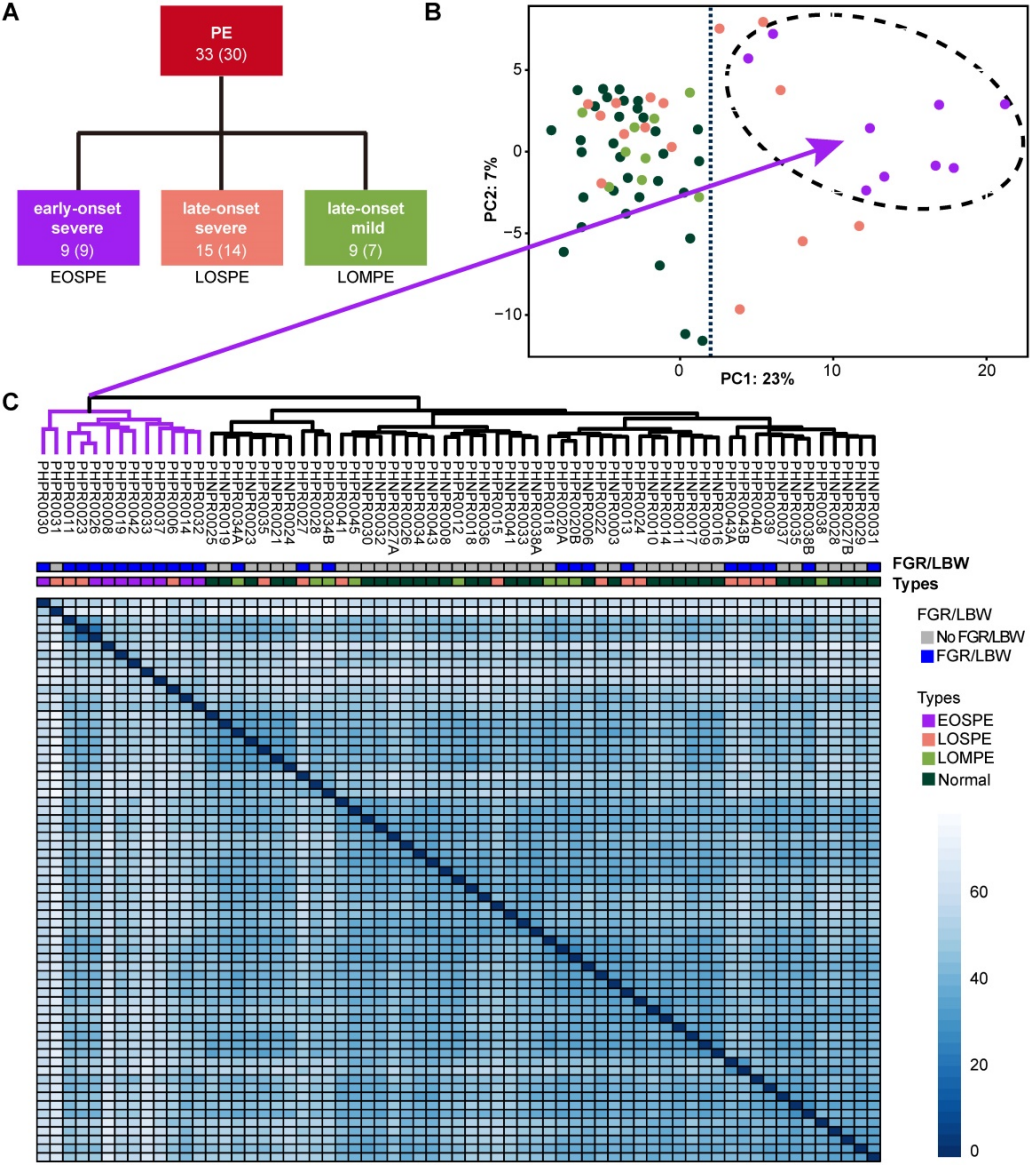
** Clinical subtypes and sample clustering using RNA-seq data. (A)** Clinical subtypes of PE patients: early-onset severe PE (EOSPE), late-onset severe PE (LOSPE), late-onset mild PE (LOMPE). The numbers under each subtype are the numbers of placentae (outside the brackets) and the numbers of patients (inside the brackets). **(B)** Principal component analysis (PCA). The top two principal components in this dataset represent up to 30% of all variations. Samples of EOSPE clustered on the right side (within the dashed circle), while samples of LOSPE and LOMPE are clustered together with normal samples on the left side. **(C)** Heatmap of sample-sample distance using the Ward's method. The EOSPE samples are clustered as the branch on the left, roughly corresponding to the samples in the right area of the PCA plot. The LOSPE and LOMPE samples are clustered together with normal samples on the right side. Four LOSPE samples were clustered with EOSPE.

We collected 32 placentae from 30 normal individuals (including 4 placentae from 2 twins) and 33 placentae from 30 PE patients (including 6 placentae from 3 twins), which were carefully classified into subtypes based on clinical characteristics (Figure 1A and Table S1). The disease group and normal group showed significant differences in systolic and diastolic pressures, delivery time and baby weights. The early-onset severe PE (EOSPE) group showed significant differences in most of these characteristics compared to the late-onset severe PE (LOSPE) cases. Both EOSPE and LOSPE showed significant differences in these characteristics compared to late-onset mild PE (LOMPE) which had only moderately elevated blood pressures compared to controls (see Table S1 for statistics and *P* values, Student's t test). Notably, the LOSPE patients had significantly elevated C-reactive protein (CRP) levels (*P* = 0.0203 between LOSPE and normal control, *P* = 0.0268 between LOSPE and EOPSE, Student's t test), whereas C-reactive protein (CRP) levels of EOPSE or LOMPE patients were within the normal range (Figure S1A). This result is consistent with previous finding that C1q and C4d are increased in the serum of LOSPE patients 2.

Four placental specimens were dissected from the midsection between the chorionic plate and maternal basal plate of each placenta soon after delivery and rapidly preserved in RNAlater® to prevent RNA degradation. RNA-seq was carried out with Illumina Highseq2500. We also performed RNA-seq on two cord-blood samples for computational removal of contamination of blood cells in the placental tissues (Table S2). Principal component analysis (PCA) and sample clustering analysis were performed on our dataset, to explore sample features in gene expression levels. The EOSPE samples were clustered together with four LOSPE samples in PCA (Figure 1B) and in the heatmap (Figure 1C). The rest LOSPE and all LOMPE samples were mixed with normal samples (Figure 1B-C). We got similar clustering results on PE samples after removing the normal samples (Figure S1B and Table S1). Looking into the clinical records, we found that the four LOSPE patients whose samples were clustered together with EOSPE samples, showed characteristics similar to those of the EOSPE patients except for gestational age at PE-onset, indicating that these four “LOSPE” patients could in fact be EOSPE patients diagnosed later than the actual onset time. We extracted the top 50 genes that contributed to the first principal component (PC1), and found that 36 are known PE-associated genes, such as *BHLHE40*,* ENG*,* FLT1*,* HK2*,* HTRA4*,* INHBA*,* LEP*,* NDRG1*,* PAPPA2*,* SASH1*,* SIGLEC6*,* SLC6A8*, suggesting that these genes may be potential molecular markers for this disease. The signatures of gene expression that separate EOSPE from the two other clinical subtypes suggest that they may have different underlying molecular mechanisms.

### Distinct dysregulated genes in PE subtypes

**Figure 2:**
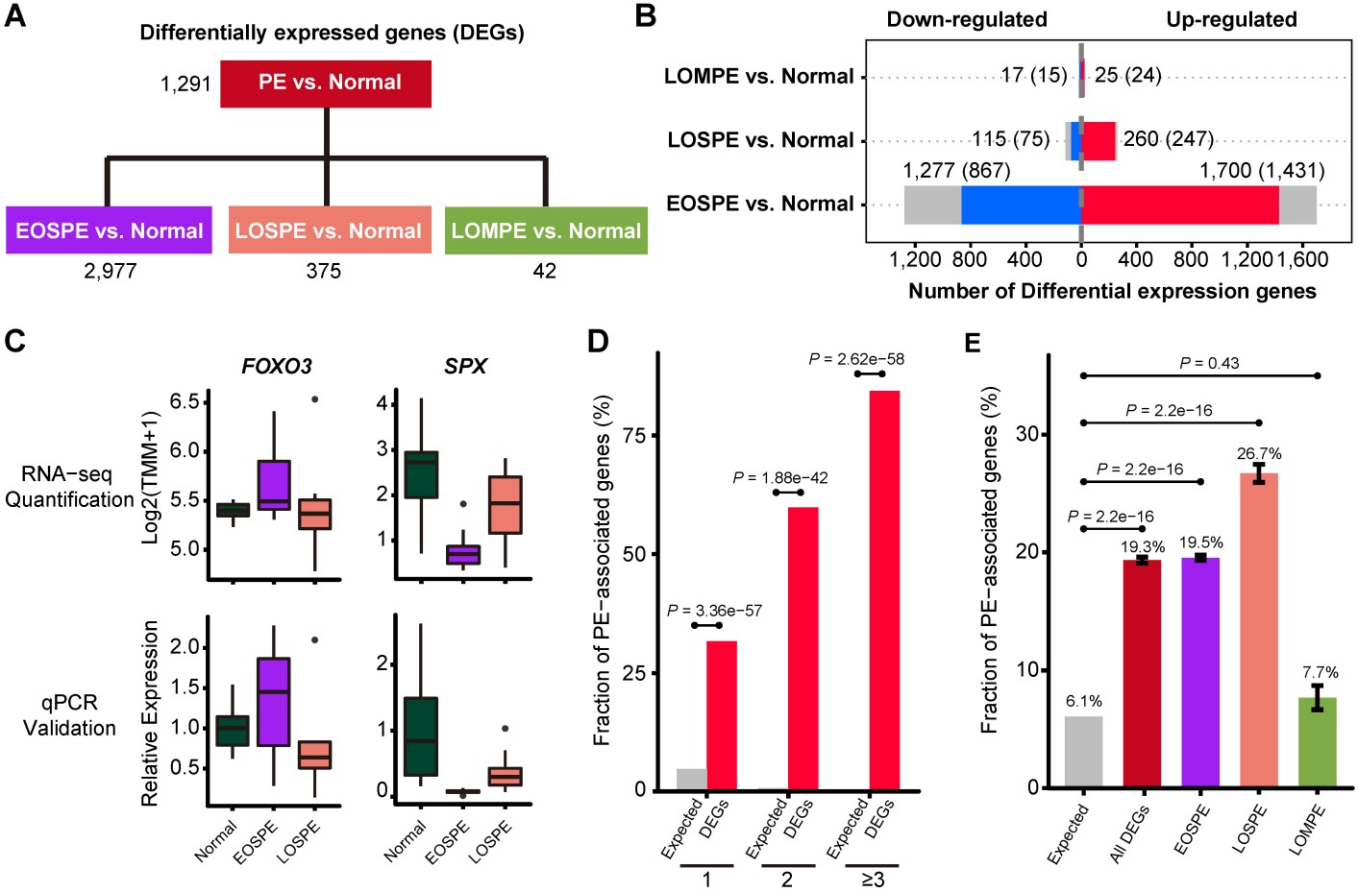
** Differentially expressed genes in preeclamptic placentae. (A)** The differentially expressed genes (DEGs) for each group were identified using two methods, DESeq2 and edgeR. The 4 comparisons of the RNA-seq data include: all PE samples vs. normal controls, EOSPE samples vs. controls, LOSPE samples vs. controls, or LOMPE samples vs. controls. **(B)** The red bars represent up-regulated genes, the blue bars represent down-regulated genes and the grey bars non-coding genes. Numbers represent DEGs in each group, and numbers in brackets represent protein-coding DEGs. **(C)** DEGs verified by qPCR. *FOXO3*: RNA-seq quantification boxplot at top-left panel and qPCR validation result at bottom-left panel. *SPX*: RNA-seq quantification boxplot at top-right panel and qPCR validation result at bottom-right panel. **(D)** The enrichment of PE associated genes in DEGs. PE-associated genes were curated from literature (Table S4). Of the genes that appear once, twice and three or more times in literature, 31.7%, 59.9% and 84.4% were recovered by the DEGs we identified in PE. The X-axis represents the number of times of a gene reported in the literature, the Y-axis represents the fraction of reported PE-associated genes under each category, with red bars representing fractions of PE-associated genes that appear once, twice, and three or more times in different literature, and gray bars the expected fractions. **(E)** The enrichment of known PE-associated genes in protein-coding DEGs in EOSPE (2,298 genes), LOSPE (322 genes), LOMPE (39 genes) and all DEGs (2,405 genes). We collected 1,177 PE-associated genes from the literature (Table S4 and Methods), and calculated the their enrichment in DEGs of different groups: red bar represents the fraction of PE-associated genes in all DEGs (19.3%, *P* = 2.2e-16), purple bar EOSPE (19.5%, *P* = 2.2e-16), pink bar LOSPE (26.7%, *P* = 2.2e-16) and light-green bar LOMPE (7.7%, *P* = 0.4261), compared to gray bar which represents the expected ratio (6.1%) of 1,177 PE-associated genes to 19,351 human coding genes (GRCH38.p12 ENSEMBL Gene V93). One-sided Fisher's exact test was used to compare the nodes kept with the nodes removed. Error bars represent the standard error of the fraction, estimated using bootstrapping method with 100 resamplings.

To identify groups of genes that may be involved in the heterogeneous pathology of the clinical subtypes, we used two different methods DESeq2 and edgeR to carry out 4 comparisons of the RNA-seq data (Figure 2B): all PE samples vs. normal controls, EOSPE samples vs. controls, LOSPE samples vs. controls, or LOMPE samples vs. controls. We found a total number of 3,100 differentially expressed genes (DEGs), including 31 microRNAs and 399 lncRNAs (Table S3).

We identified 2,977 DEGs (2,298 protein-coding genes) by comparing EOSPE samples with controls, while we only got 1,291 DEGs by comparing all PE samples with controls, suggesting that a genetic homogeneity within the EOSPE cases could have increased the detection power. For the other two subtypes, we found 375 DEGs (322 protein-coding genes) between LOSPE samples and controls, and 42 DEGs (39 protein-coding genes) between LOMPE samples and controls (Figure 2A and Figure S2A, and Table S3). The large differences between the numbers of DEGs in the three clinical subtypes (EOSPE, LOSPE and LOMPE) indicate that there are different molecular mechanisms underlying the clinical subtypes (Figure 2B). In general, we found more up-regulated genes than down-regulated genes. The numbers of up-regulated genes are 1.3~2.3 times higher than those of down-regulated genes in the three comparisons. We carried qPCR to validate some of the differentially expressed genes (Figure 2C, Figure S2B-C).

To assess the functional relevance of our dataset, we compared our DEGs with 1,177 PE-associated genes collected from the literature (Table S4). Most of the reliable PE-associated genes which appear twice or more in literature are recovered in our dataset (Figure 2D). Of the 1,177 known PE-associated genes, the DEGs in our dataset (protein-coding genes) recover 31.7% (296/934), 59.9% (88/147) and 84.4% (81/96) of those found once, twice, three or more times in literature. The high consistence of our data with well-reported PE-associated genes suggest high quality of our dataset. These PE-associated genes show significant enrichment in DEGs (protein-coding genes) of EOSPE and LOSPE: 19.3% (*P* = 2.2e-16, One-sided Fisher's exact test) in all DEGs, 19.5% (*P* = 2.2e-16, One-sided Fisher's exact test) in DEGs of EOSPE, 26.7% (*P* = 2.2e-16, One-sided Fisher's exact test) in DEGs of LOSPE, and 7.7% (*P* = 0.4261, One-sided Fisher's exact test) in DEGs of LOMPE (Figure 2E).

Because PE has the most significant consequence on developmental brain impairments in offspring, which lead to intellectual disability 17, epilepsy 18, autism 19-21 and schizophrenia 22, 23, we performed the enrichment analyses of schizophrenia-associated genes in DEGs, and found that the disease-associated genes were significantly enriched (Figure S2D). These results suggest that the differentially expressed genes are not only associated with PE, but also with consequence of PE on the offspring in their later life.

### Molecular pathways involved in PE

**Figure 3:**
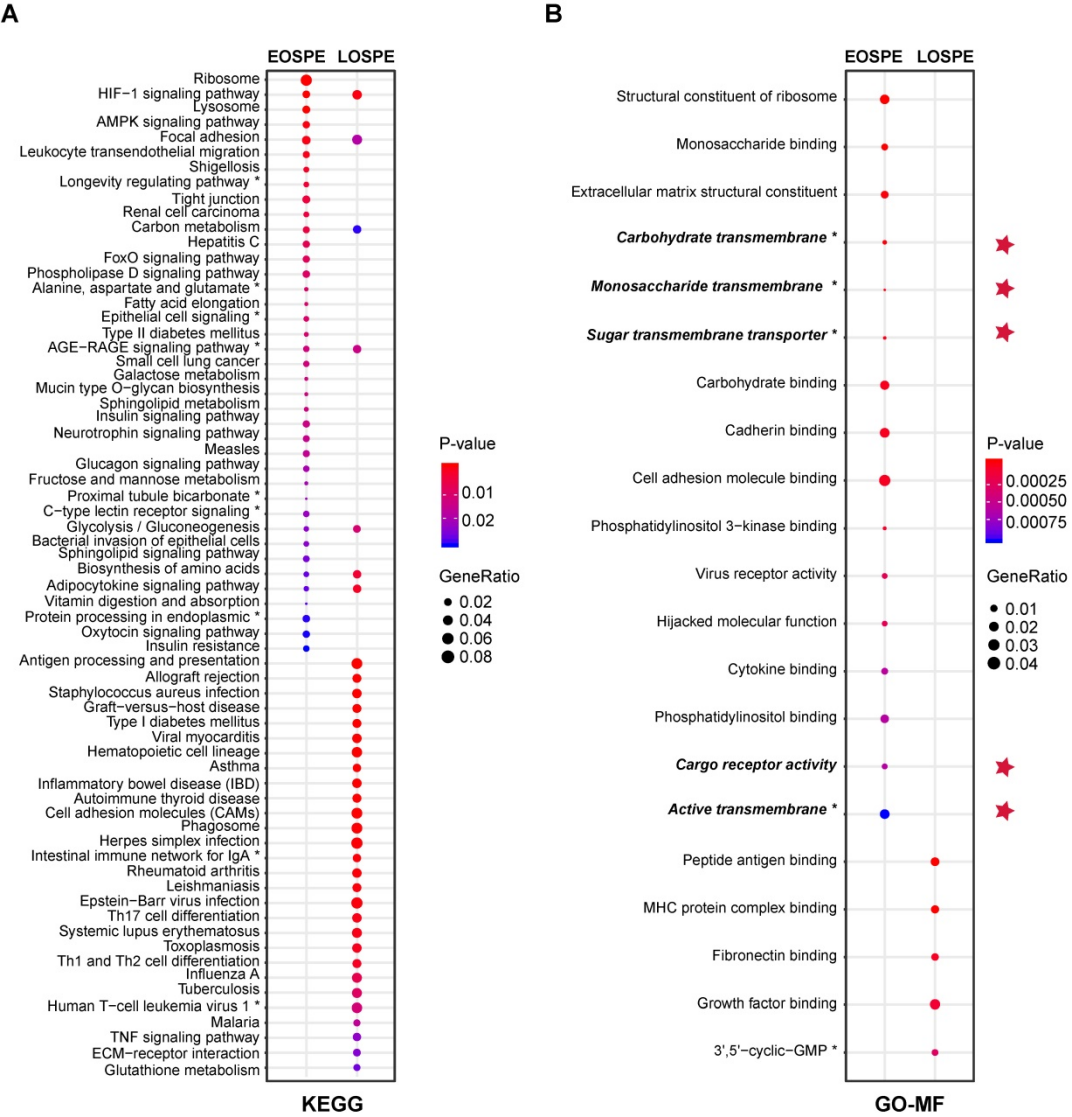
** Enrichment of KEGG pathways and GO molecular function (MF) terms with differentially expressed protein-coding genes of EOSPE and LOSPE. (A)** Enriched KEGG pathways with protein-coding DEGs of EOSPE and LOSPE. To save figure space, “*” is used to label the shortened terms, and the complete terms are: (1) Longevity regulating pathway - multiple species (2) Alanine, aspartate and glutamate metabolism (3) Epithelial cell signaling in Helicobacter pylori infection (4) AGE-RAGE signaling pathway in diabetic complications (5) Proximal tubule bicarbonate reclamation (6) C-type lectin receptor signaling pathway (7) Protein processing in endoplasmic reticulum (8) Intestinal immune network for IgA production (9) Human T-cell leukemia virus 1 infection. **(B)** Enriched GO MF terms with protein-coding DEGs of EOSPE and LOSPE. Dots represent the enriched KEGG pathways or GO MF terms with description of each pathway and term; colors represent scale of *P*-values, the sizes of dots represent ratio of DEGs in corresponding pathways and GO terms, and red stars indicate transporter terms in GO MF. To save figure space, “*” is used to label the shortened terms, and the complete terms are: (1) Carbohydrate transmembrane transporter activity (2) Monosaccharide transmembrane transporter activity (3) Sugar transmembrane transporter activity (4) Active transmembrane transporter activity (5) 3',5'-cyclic-GMP phosphodiesterase activity. Detailed information is listed in Table S5.

The large differences between DEGs observed in EOSPE and LOSPE suggest that these two major subtypes may have different molecular mechanisms. To explore the associated pathways and molecular functions of the DEGs of these two major subtypes, we performed enrichment analyses on the Kyoto Encyclopedia of Genes and Genomes (KEGG) and Gene Ontology (GO) terms for the protein-coding DEGs of EOSPE (2,298) and LOSPE (322) (Figure 3). We found that 39 enriched pathways for EOSPE and 35 enriched pathways for LOSPE (Table S5). Importantly, the enriched pathways for EOSPE and LOSPE were largely distinct. Most of the enriched pathways of EOSPE were involved with metabolism, such as lysosomal function, AMPK signaling, tight junction pathways, insulin signaling, fatty acid elongation, galactose metabolism, alanine, aspartate and glutamate metabolism pathways (Figure 3A), many of which are reported to be associated with PE in previous studies (Table S5). By contrast, most of the enriched pathways of LOSPE were involved in immune or autoimmune pathways, such as allograft rejection, inflammatory bowel disease, rheumatoid arthritis, HTLV-I infection and tuberculosis pathways (Figure 3A). The immune genes *HLA-DRB1*, *HLA-DQA1*, *HLA-DRA*, *HLA-DPA1*, *HLA-DPB1* and *HLA-DMB* were up-regulated in LOSPE, but not in EOSPE. These results suggest that the two main subtypes EOSPE and LOSPE may have different underlying mechanisms. The subtype EOSPE may be mainly caused by disturbance in metabolism, while the subtype LOSPE by dysfunction of the immune system in the placenta. The metabolic disturbances in EOSPE and the abnormal immune functions in LOSPE may lead to changes in other related pathways in the placenta. We obtained similar results in the pathway enrichment analysis for DEGs of EOSPE-only, LOSPE-only and shared DEGs between the two subtypes (Figure S3A).

We further performed pathway enrichment analysis for up-regulated and down-regulated DEGs respectively, and found that the up-regulated genes and down-regulated genes were involve in different pathways in EOSPE (Figure S3A-B, and Table S5). Very interestingly, synapse-related pathways (such as 'Morphine addiction', 'Nicotine addiction and 'GABAergic synapse') and pathways related to synaptic plasticity (such as 'cGMP-PKG signaling pathway', 'Retrograde endocannabinoid signaling' and 'Circadian entrainment' are enriched down-regulated DEGs of EOSPE (Figure S3A-B, and Table S5). No enriched pathway for down-regulated genes in LOSPE (Figure S3A and C, and Table S5).

We also performed GO-term enrichment analysis, and found that the DEGs in EOSPE were involved in distinct molecular activities (GO-MF) from those in LOSPE (Figure 3B). We detected significant enrichment of DEGs of EOSPE in 16 molecular function terms of GO, including key molecular activities in constituents of ribosomal structure, differential sugar binding and transmembrane transporter activities, phosphatidylinositol binding, adhesion-molecule binding, virus receptor activities and molecular functions hijacked by virus (Figure 3B). We detected significant enrichment of DEGs of LOSPE in only 5 molecular function terms of GO, three of which are involved in the immune response, and the other two terms are growth factor binding and 3',5'-cyclic-GMP phosphodiesterase activity (Figure 3B). These results are consistent with the results of pathway enrichment analysis we showed above (Figure 3A): the major molecular disturbance in the placenta of EOSPE may be involved in abnormal metabolism, while predominated functional impairment in LOSPE may be involved in immune-function abnormalities. The results of enrichment analyses on biological process (BP) terms and cellular component (CC) terms of GO support the same conclusion (Figure S3D-E, and Table S5).

### Transporter genes involved in PE

**Figure 4:**
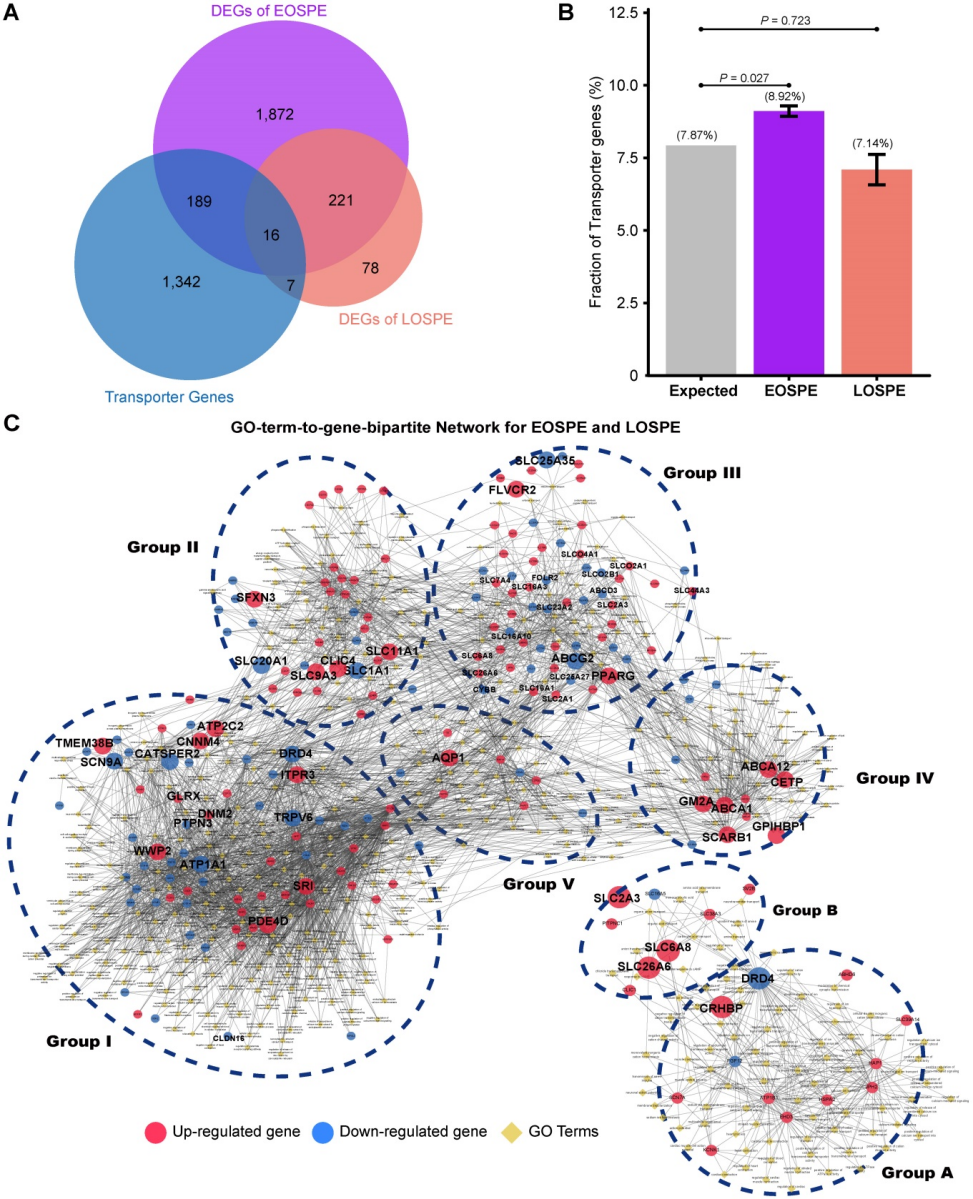
** Functional classification of differentially expressed transporter genes. (A)** The overlapping genes between the transporter genes and DEGs. Of the identified transporter genes, 205 were differentially expressed in EOSPE and 23 genes in LOSPE. **(B)** Fraction of transport genes in DEGs. The transporter genes were collected from Gene Ontology. Gray bar represents the expected fraction (7.87%) of transporter genes in all GO annotated genes (19,738), the fraction of transporter genes in the DEGs of EOSPE samples (8.92%), the DEGs of LOSPE (7.14%). The transporter genes were significantly enriched in the DEGs of EOSPE, but not in LOSPE. One-sided Fisher's exact test was used to calculating the *P*-values. Error bars represent the standard error of the fraction, estimated using bootstrapping method with 100 resamplings. **(C)** Gene-GO term bipartite network for enriched GO BP terms and DEGs in EOSPE and LOSPE. Blue dashed circles show 5 biological process clusters involved in different transportation processes of substrates and materials. Group I: ion channels; group II: macro-autophagy; group III: transmembrane transportation of nonlipid nutrients; group IV: transmembrane transportation of lipids; group V in transportation of hormone. In the LOPSE, the group A: ion channels; group B: transportation of chloride, carboxylic acid, cAMP and anion. Blue dashed circles show 2 biological process clusters. Red nodes represent up-regulated transporter genes and blue nodes down-regulated transporter genes, and yellow diamond nodes represent GO BP terms. The edges indicate the GO BP terms the genes belong to.

Because the DEGs of EOSPE were enriched in many transmembrane transporter activities of GO MF terms (Figure 3B), we collected 1,554 transporter genes by searching The Gene Ontology (GO) database with key words “transporter”, “carrier” or “substrate exchanging” in order to further look into the dysregulated molecular transportation in the placenta of EOSPE patients (Table S6). We found 13.2% (205 of 1,554) transporter genes in DEGs of EOSPE, and only 1.5% (23/1,554) transporter genes in DEGs of LOSPE (Figure 4B and Table S6). Notably, these transporter genes were significantly enriched in the DEGs of the EOSPE (*P* = 0.027, One-sided Fisher's exact test), but not in LOPSE (*P* = 0.723, One-sided Fisher's exact test) (Figure 4B).

The 205 transporter genes in DEGs of EOSPE were enriched in 397 GO biological process (BP) terms (Adjusted *P* ≤ 0.05, hypergeometric test from ClusterProfiler Package). For these genes, we constructed a GO-term-to-gene bipartite network for all enriched BP terms, which contained 5 groups of functionally related genes (Figure 4C and Table S7). Group I contains genes involved in different ion channels, some of which were previously reported to be associated with PE, e.g. *ATP1A1*,* CACNA1D*,* CALM1*,* EHD3*,* FGF12*,* KCNE1*,* KCNH5*,* NOS1AP*,* PDE4D*,* SCN1B*,* SCN4B* and* SRI* (Table S7); group II contains genes involved in macro-autophagy, which was also previously proposed to be involved in PE 51, 52, such as some up-regulated genes *ATP6V1B1*,* ATP6V1C2*,* ATPV1D1*,* ATP6V0A1*,* ATP6V0E1*, and *ATP6V0D1;* group III, genes in transmembrane transportation of hexose, fatty acids, amino acids, vitamins, organic anions and mitochondrial components; group IV, genes in transmembrane transportation, storage or homeostasis of lipids, sterols, cholesterol and phospholipids, and group V, genes in transportation of hormone, response of cAMP and drug, secretion of insulin and protein. (Figure 4C). The detailed information of all five groups is included in the Supplemental material (Table S7). We also performed pathway enrichment analysis for these 205 differentially expressed transporter genes in EOSPE and found that these genes were enriched in 34 pathways (Figure S4 and Table S7), which are consistent with the five functional groups in GO-term analysis (Figure 4C). Some of these pathways are known to be involved in PE, such as oxidative phosphorylation 43, ABC transporter pathway 53 and circadian entrainment pathway 54.

The 23 transporter genes in the DEGs of LOSPE were enriched in 93 GO biological process terms (Adjusted *P*-value ≤ 0.05, hypergeometric test from ClusterProfiler Package). We also constructed a GO-term-to-gene-bipartite network for these 93 terms, which contained 2 groups of functionally related genes (Figure 4D). The group A contains 15 genes involved in ion channels, and group B only contains 8 genes involved in transportation of chloride, carboxylic acid, cAMP and anion.

The placenta serves as a molecular barrier between maternal and fetal circulation, and transmembrane transporters play a key role in the placental exchange function. On one hand, the fetus receives nutrients from the mother, such as carbohydrates, lipids, amino acids, vitamins and minerals for adequate development and growth, and on the other hand, the fetus can drain waste products via efflux processes into the maternal circulation 55. The dysregulation of the quantity, density and activity of membrane transporters could impact the nutrient supply and consequently affect fetal development. We consistently observed low birth weight (LBW) and fetal growth restriction (FGR) in 90% (9 of 10) of the babies born by EOSPE patients and 53% (8 of 15) of the babies born by LOSPE patients (Table S1). The weights of babies in EOSPE group were significantly lower than those of babies in LOSPE group (Table S1).

### Key regulators involved in PE

**Figure 5:**
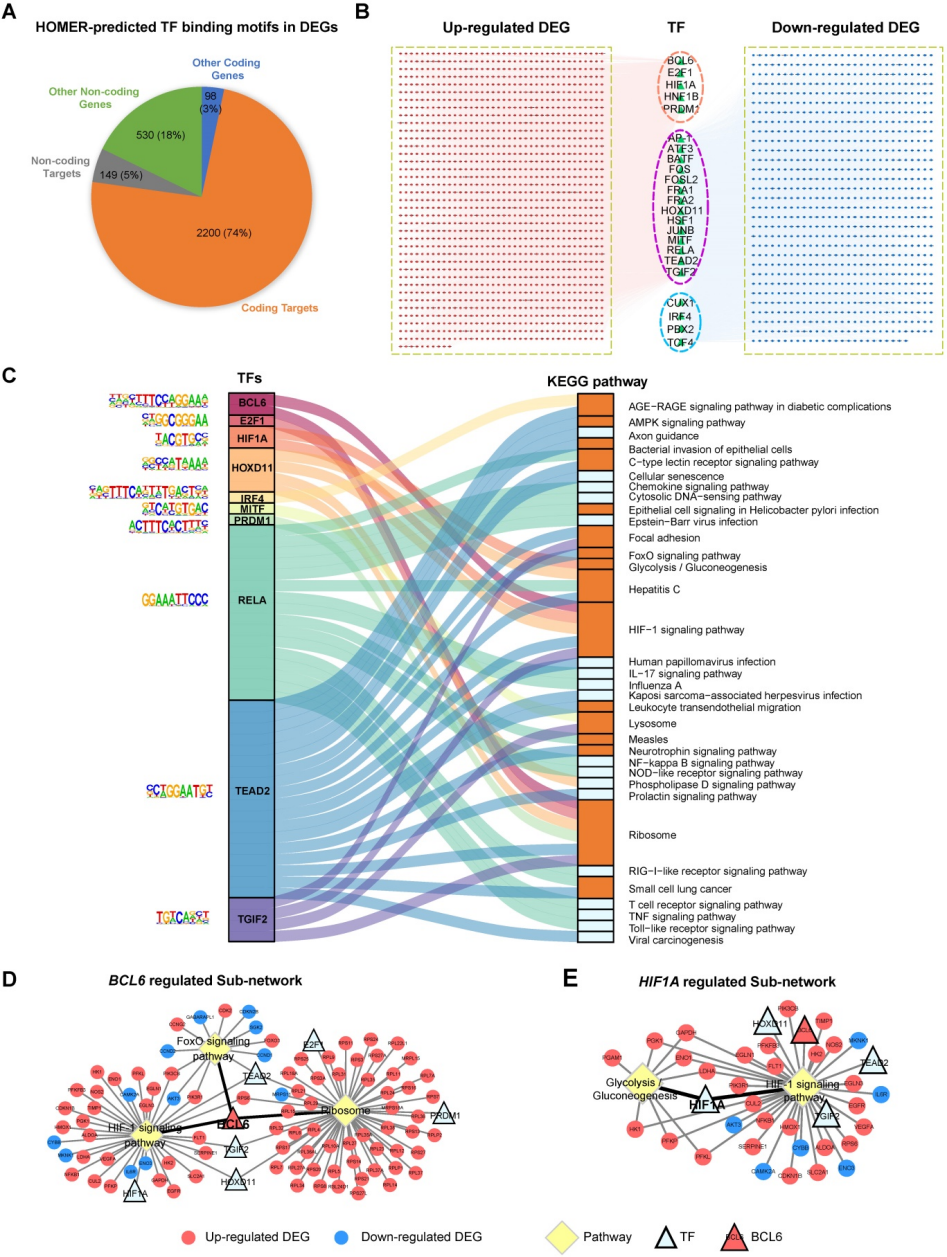
** Identification of critical transcription factors that contribute to the dysregulation of pathways involved in EOSPE. (A)** Transcription factor-binding motifs were searched in the DEGs of EOSPE using HOMER. Of 2977 DEGs in EOSPE, 79% (2349/2977) were predicted to be targeted by 23 TFs.** (B)** TF-targets network for the DEGs of EOPSE. Of the 23 enriched TFs (the triangles), 5 TFs (pink circles) target the up-regulated DEGs, 4 TFs (blue circles) target the down-regulated DEGs, and 14 TFs (purple circles) target the both up- and down-regulated DEGs. The up-regulated targets are in the red nodes (1454), and the down-regulated targets are in the blue nodes (895).** (C)** The Sankey diagram showing the relationship between TFs and the enriched pathways with its targets. Enrichment analysis was performed on the targets of each TF. A total number of 34 enriched pathways (the right column) were found for 10 TFs (colored column in the left). The binding motifs corresponding to TFs are listed on the left. The orange color in the right bar indicates the pathways that are overlapped with those enriched with DEGs of EOSPE (Figure 3A), and the light blue color in the right bar indicates the pathways that are newly found enriched pathways with the targets of TFs.** (D-E)** 'TF-Target-Pathway bipartite networks' for TFs *BCL6* and *HIF1A*. Most of *BCL6* targeting DEGs are involved in three KEGG pathways **(D)**. The *HIF1A* targeting DEGs are involved in two pathways **(E)**. Circles: non-TF genes; triangles: TFs; yellow diamonds: KEGG pathways; red: up-regulation; blue: down-regulation; light blue: no expression change.

We want to know if the distinct differential gene expression patterns in EOSPE and LOSPE might be due to the activity of a set of transcription factors (TFs). We predicted TF-binding motifs for the DEGs of EOSPE and LOSPE using HOMER, and obtained 23 and 9 enriched motifs for the two sets of DEGs. In EOSPE, 23 motifs were found in 2,359 DEGs (78.90%) including 2,200 protein-coding genes (95.74%). In LOSPE, 9 motifs were found in 301 DEGs (80.27%) including 289 protein-coding genes (89.7%) (Figure 5A and Figure S5A). After manually checking human genes with enriched motifs, we constructed the TF-targets networks for the EOSPE and LOSPE (Table S8). TFs such as *BCL6*, *E2F1*, *HIF1A*, *HNF1B* and *PRDM1* target the up-regulated DEGs EOSPE, and TFs such as *CUX1*, *IRF4*, *PBX2* and *TCF4* target the down-regulated DEGs in EOSPE, while the other 14 TFs target both up-regulated and down-regulated genes (Figure 5B). In LOSPE, *HOXD11*, *IRF1*, *LHX3*, *NKX2*-5, *ONECUT1* and *SOX17* target upregulated genes, and no TFs target the down-regulated DEGs (Figure S5B). Moreover, TFs such as *BCL6*, *ATF3*, *BATF*, *FOSL2* also show significant differential expression in the EOSPE. Some of these TFs are well documented to be involved in preeclampsia. For example, *BCL6* is reported to associate with PE in multiple studies 56, 57, and *HIF1A* is also well known to be involved in PE 58, 59. These results suggest that only small number of TFs may regulate many functional genes in PE.

We further searched enriched biological pathways for the differentially expressed targets of each TF. Of the 23 TFs found for DEGs of EOSPE, 10 TFs have a total number of 39 enriched KEGG pathways. We constructed a Sankey diagram to illustrate the TFs and their potential downstream pathways, showing a potential collaborative regulation between these TFs (*P* ≤ 0.05, Figure 5C and Table S8). For example, *BCL6* and *HIF1A* collaboratively may regulates 'HIF1 signaling pathway', which is well known to be involved in PE 60, 61. *BCL6*, *E2F1*, *HOXD11* and *PRDM1* may collaboratively regulate 'ribosome pathway', which is another critical pathway in the pathogenesis of PE 62, 63 and is the most significantly dysregulated in EOSPE (Figure 3A). We constructed 'TF-Target-Pathway bipartite networks' for each TF (Figure 5D-E and Figure S6). *BCL6* regulates the differentially expressed targets of three critical pathways for PE (Figure 5D). *HIF1A* regulates two critical pathways for PE (Figure 5E). These results suggest that further studies on these 10 TFs and their targeting pathways would possibly illustrate some mechanisms underlying EOSPE. Of the 9 TFs identified in LOSPE, 7 TFs have 32 enriched KEGG pathways (*P* ≤ 0.05, Table S8). The Sankey diagram for TFs and their potential downstream pathways shows a collaborative regulation between TFs (Figure S5C). We further constructed 'TF-Target-Pathway bipartite networks' for TF *HOXD11* and *SOX21*. The *HOXD11* may regulate 19 pathways and *SOX21* two pathways. These pathways are related to immune and inflammation functions (Figure S5D-E). These results imply that a small number of key TFs may largely determine the pathway dysregulation in the placenta of both EOSPE and LOSPE.

## Discussion

Patients with PE display a wide range of symptoms. Early-onset PE patients usually have severe symptoms; among the late-onset PE patients, however, some show severe symptoms, while others are only mildly affected. Although the clinical characteristics of PE can be measured, the underlying molecular bases for the clinical subtypes are not known. Previous studies on placentae of PE patients only found a small number of dysregulated genes due to small sample sizes and no distinguishing of clinical subtypes 41, 46, 51. These studies point to some abnormal molecular processes, such as defective angiogenesis 64, 65, hypoxia 59, 66, 67 and inflammation 42, 68, 69, but they have not unambiguously define the different molecular pathways involved in the clinical subtypes. A study on classified samples based on clinical characteristics are needed to identify the genes underlying the differential pathogenesis of the PE subtypes. In order to systematically search for genes involved in clinical PE subtypes, we collected placental tissues between the chorionic and maternal basal surfaces of placentae from patients with classical presentation of preeclampsia, which were grouped into three clinical subtypes: early-onset severe PE (EOSPE), late-onset severe PE (LOSPE) and late-onset mild PE (LOMPE) (Table S1). Our identification of the distinct molecular processes involved in the clinical subtypes was based on initial classification and comparison of RNA-seq data of the well classified samples. Because we noticed that the blood could not be removed from placental tissues during sample collection, which might be one factor affecting previous transcriptomic analyses, we removed cord-blood contamination using a computational method based on RNA-seq data of whole cord-blood samples (Table S2).

In the principal component analysis (PCA) and sample clustering analysis, the EOSPE samples clustered together, separating themselves from LOSPE and LOMPE samples (Figure 1B). We divided the 33 PE samples into three groups (EOSPE, LOSPE and LOMPE) for transcriptome analysis, and carried out analyses on both divided and undivided samples and found that analysis on divided samples gave better results (Figure 2A). Although dividing samples by subtypes resulted in smaller sample size which might affect the analysis, we obtained better results on divided samples probably due to higher genetic homogeneity. We carried out comparisons between the placental transcriptomes of the three clinical subtypes and normal samples (EOSPE vs. normal, LOSPE vs. normal and LOMPE vs. normal) to identify the differentially expressed genes in the three clinical subtypes. Surprisingly, EOSPE had 2,977 differentially expressed genes (2,298 protein coding genes), while LOSPE had only 375 (322 protein coding genes), indicating different molecular mechanisms for these two subtypes (Figure 2A); both data sets, however, showed significant enrichment of previously reported PE-associated genes (Figure 2C). Moreover, LOMPE had only 42 differentially expressed genes that showed no enrichment for the previously reported PE-associated genes (Figure 2C), suggesting a lesser degree of involvement of genetic factors in the pathogenesis of mild PE. Although the pregnancies of all EOSPE patients and some LOSPE patients were terminated before full-term delivery, which might affect the genes expression in the placenta, the DEGs between PE placentae and normal subjects are validated by the PE-associated genes from literature (Figure 2D). Most interestingly, we found distinct molecular pathways involved in the two major subtypes of PE: metabolisms and related activities in the EOSPE and immune activities in LOSPE (Figure 3). More enrichment of immune pathways in LOSPE is in consistent with the increased C-reactive proteins (Figure S1A) and increased C1q and C4d in the serum of LOSPE patients 2. However, different from previous findings that EOSPE is impacted by immune imbalance 2, our results suggest that EOSPE are associated with dysregulated metabolic pathways. These PE-associated pathways were predicted to be regulated by 23 transcription factors in EOSPE and 9 TFs in LOSPE (Figure 5), which might be the driver genes during the abnormal development of the placenta of PE patients. Some of these transcription factors such as *BCL6* and *HIF1A* are well known PE candidate 56-58. These small number of key regulators collaboratively regulate the downstream pathways through modifying gene transcription. For example, *BCL6* and *HIF1A* may together regulates 'HIF1 signaling pathway', a pathway well known to be involved in PE 56-61, 70. *BCL6*, *E2F1*, *HOXD11* and *PRDM1* may cooperatively regulate 'ribosome pathway', which is the most significantly dysregulated in EOSPE (Figure 3A) and reported to be a critical pathway in the pathogenesis of PE 62, 63. Therefore, further investigating these key regulators may help illustrate the molecular mechanisms of PE.

As the placenta is the supporting system for a developing embryo, the widespread gene expression change in placentae of PE patients might have profound effects on the development of the fetuses and the health of the offspring in later life. Accumulating evidences point to association of preeclampsia with brain developmental disorders, including intellectual disability 17, epilepsy 18, autism 19-21 and schizophrenia 22, 23. It might be partially due to the placenta-derived factors that affects the development of the brain and thus leads to complex brain disorders 23, 71. Ursini et al found that, for some loci of schizophrenia, the association between genetic risk and schizophrenia is affected by complicated pregnancies, and the gene set within these loci are differentially expressed in placentae from complicated in comparison with normal pregnancies 23. In order to investigate to what extent the differentially expressed genes in PE may be also involved in schizophrenia, we collected 3,437 schizophrenia-associated genes from classical schizophrenia databases and literature, and found that the schizophrenia-associated genes are significantly enriched in DEGs of EOSPE (*P* = 1.4e-4, One-sided Fisher's exact test) and LOSPE (*P* = 0.01, One-sided Fisher's exact test) (Figure S2D). The synapse-related pathways, such as 'GABAergic synapse', and pathways involved in synaptic plasticity, such as 'cGMP-PKG signaling pathway', are enriched down-regulated DEGs of EOSPE, but not in LOSPE (Figure S3A-B, and Table S5), suggesting that EOSPE might increase the risk of psychiatric disorders in the offspring.

Taken together, this study provides molecular-level evidences that EOSPE and LOSPE are two different subtypes with distinct underlying molecular mechanisms, while LOMPE may not be due to placental factors. Expression of metabolism-related genes is significantly affected in EOSPE, whereas in LOSPE it preferentially involves the expression of immune-related genes. Notably, the transporter genes dysregulated in EOSPE may cause abnormal molecular exchange between maternal and fetal circulation, leading to fetal growth restriction. The predicted TF-binding motifs in differentially expressed genes demonstrate that only a small number transcription factors may serve as driving genes, causing differential expression of a larger number of genes in EOSPE and LOSPE. The key regulators collaboratively may regulate critical pathways involved in EOSPE and LOSPE. These findings provide basis for further studying the distinct mechanisms underlying two major PE subtypes, which could potentially lead to more effective therapies to target different subtypes.

## Methods

### Sample collection and criteria for grouping PE cases into different clinical subtypes

We collected the tissue samples at the Department of Obstetrics & Gynecology of Nanfang Hospital in China from January 2015 to July 2016. The clinical characteristics of each patient were extracted from the medical records, which strictly followed the American Board of Obstetrics and Gynecology, Williams Obstetrics 24th edition. Samples of placental tissues were collected from the mid-section placental tissues of the placenta, between the chorionic and maternal basal surfaces, at four different positions within 5 minutes after delivery. These placental tissues were placed into RNAlater® solution and stored at -80 °C. This research has been approved by the Ethnics Board of Nanfang Hospital of Southern Medical University, and all patients have signed the informed consent.

The diagnostic criteria for preeclampsia (PE) were: new-onset hypertension (systolic blood pressure ≥ 140 mmHg and/or diastolic blood pressure ≥ 90 mmHg) on at least 2 occasions at least 4 hours apart after 20 weeks of gestation, accompanied by one or more of the following features: proteinuria (≥ 0.3 g/24 hours or more, or ≥ 2+ on dipstick analysis of urine), maternal organ dysfunction (including renal, hepatic and neurological), or hematological involvement such as thrombocytopenia, and/or uteroplacental dysfunction, such as fetal growth restriction 1, 72. The “preeclampsia with severe features” or briefly “severe PE” was diagnosed if patients with PE have systolic blood pressure ≥160 mmHg and/or diastolic blood pressure ≥110 mmHg on 2 occasions at least 4 hours apart while the patients is on bed rest, accompanied by one or more of the following symptoms: significant proteinuria of ≥ 5g/24 hours or ≥ 3+ on urine dipstick, liver function deterioration, thrombocytopenia (platelet count < 100,000/microL), oliguria (≤ 500 ml in 24 hour), creatinine ≥ 1.1 mg/dL or a doubling of the serum creatinine, cerebral or visual disturbances 73. According to gestational age at its diagnosis, PE can be classified into early-onset (< 34 weeks) and late-onset (≥ 34 weeks) 74, 75. Although other gestational age cut-offs have been suggested, 34 weeks remains the most commonly used 76, 77, presumably as the rate of neonatal morbidities declines considerably after reaching this time point. The date of onset was defined as the gestational age when both blood pressure and proteinuria criteria were first diagnosed. All women delivered by C-section without labour were included. Exclusion criteria included pregnancies in women with a previous history of essential hypertension (chronic hypertension), type-I or type-II diabetes, thyroid insufficiency, cardiovascular disease, chronic inflammatory, or chronic renal disease, hepatitis, and chorioamnionitis. The pregnancies with gestational hypertension and/or preterm delivery (before 37 weeks + 0 days of pregnancy) were considered as exclusion criteria for the controls. Other exclusion criteria included consecutive miscarriages (≥ 2 pregnancy losses) and/or fetal anomaly. Considering that the patients with twin pregnancies did not show more severity of PE symptoms, the 9 pregnancies (4 with PE and 5 normal) with twins were not excluded from both controls and PE samples.

Based on the criteria described above, we grouped our PE patients into three clinical subtypes: (1) early-onset severe PE (EOSPE): new-onset hypertension (systolic blood pressure ≥160 mmHg and/or diastolic blood pressure ≥110 mmHg) with significant proteinuria ( ≥ 5g/24 hours or 3+ on urine dipstick) before 34 weeks of gestation, and with one or more severe features (such as liver function deterioration, thrombocytopenia); (2) late-onset severe PE (LOPSE): similar symptoms as EOSPE, but new-onset after 34 weeks of gestation; (3) late-onset mild PE (LOMPE): new-onset hypertension accompanied proteinuria after 34 weeks of pregnancies without severe features described above. The summary of clinical characteristics of these three clinical subtypes and comparisons between the subtypes were listed in Table S1.

### RNA-seq

Total RNA was isolated using the RNeasy® Plus Universal Mini Kit (Qiagen) according to the manual protocol. RNA-seq was carried out at Berry Genomics Corporation (Beijing, China). Briefly, RNAs with polyA tails were isolated, and double-stranded cDNA libraries were prepared using TruSeq RNA Kit (Illumina) followed by paired-end sequencing using Illumina Hiseq 2500. In order to computationally remove the cord blood contamination, RNA-seq was also done on two samples of cord blood. The reads from the clean data of RNA-seq were aligned to the human reference genome (hg38) with STAR 78 and counted using HTSeq 79 with union mode. Raw counts for annotated genes (protein coding genes, rRNA, microRNA, LncRNA, pseudogenes and so on) in the General Transfer Format (GTF) annotation file were obtained. The reference genome sequence and GTF annotation files were downloaded from the Ensembl website (http://asia.ensembl.org/index.html) in the alignment and counting reads steps.

Annotation information, such as gene location, gene types, gene symbols, for differential expression genes were extracted from EnsDb.Hsapiens.v86 package. Four brief categories of gene biotypes were divided following the table listed on the GENCODE website page (https://www.gencodegenes.org/gencode_biotypes.html). Gene Ontology terms overrepresentation, Disease Ontology, Pathway and functional annotation analyses were used clusterProfiler 80 and DOSE 81 packages. All analyses were done in R-platform, and all packages and public datasets were listed in Table S9.

### Computational removal of blood contamination

The contaminated cord blood cells could not be totally washed from the placenta tissue samples, so the extracted RNAs contained some from cord blood cells. To better study the difference in gene expression of placenta tissues between PE patients and controls, we removed the contamination using the expression level of a cord blood marker gene for normalization. Cord blood samples from two normal pregnancy women were sequenced using the same RNA-seq method. Differentially expressed gene analysis was applied to two cord blood samples against two normal placenta samples to identify marker genes for cord blood cells. We used the Hemoglobin Subunit Mu (*HBM*), a subunit of hemoglobin that is specifically expressed in red blood cells, and recalibrate the raw count of genes accordingly in the following equation:

geneX_actual_ = geneX_placenta_ - geneX_cord blood_ × (HBM_placenta_/HBM_cord blood_)

The raw counts of genes in each sample before and after removing blood contamination were showed in Table S2.

### Clustering analysis of PE samples only

The EOSPE samples were separated from other samples in clustering, whereas LOMPE and LOSPE samples were clustered together with normal samples (Figure 1B). To explore clusters of PE samples, we performed clustering analysis on PE samples only, including EOSPE, LOSPE and LOMPE samples, excluding normal samples. The top 500 most variances (genes) across all PE samples were used for clustering (Figure S1B). All of the LOMPE samples and most of the LOSPE samples clustered together on the left side of the graph as cluster-1, whereas all EOSPE samples clustered on the right side as cluster-2, together with the four LOSPE samples. In addition, we carried out a comparison of the clinical characteristics of the patients between the two clusters and detected significant differences in blood pressure, gestational age of delivery, baby weight, FGR or LBW and proteinuria (Table S1). Of the 14 babies (13 placenta samples) in subclass-2, 12 babies (85.7%) had FGR or LBW, whereas 10 of 20 babies (50%) had FGR or LBW in subclass-1. The mean length of gestation in two clusters were 264 (37.7 weeks) and 228.8 (32.5 weeks), and patients in the two clusters showed different weeks at onset of PE. The quantification of proteinuria in the two clusters also had significant differences. In other words, PE samples in cluster-1 showed late-onset, relatively moderate symptom, and less harmful outcomes; PE samples in cluster-2 showed early-onset, more severe symptom, and worse outcomes.

### Methods for identifying differentially expressed genes (DEGs)

Two widely used tools in R platform, DESeq2 82 and edgeR 83, were applied to determine differential expression genes in different comparisons. The genes with at least 1 count in every samples were used in DESeq2 method and the genes with CPM (Counts Per Million) value larger than 1 in at least one sample were used in edgeR method.

Samples similarity in each group was calculated using all information of expressed genes (raw count of gene in all samples is more than 1) for heat-map, and top 500 genes with the biggest variations were used for PCA analysis. Four comparisons between the clinical subtypes of PE patients were carried out using both DESeq2 and edgeR: **(i)** all PE placental samples (n = 33) versus all normal placental samples (n = 32); **(ii)** late-onset mild PE placental samples (n = 9) versus all normal placental samples; **(iii)** early-onset severe PE placental samples (n = 9) versus all normal placental samples; and **(iv)** late-onset severe PE placental samples (n = 15) versus all normal placental samples (Figure 2). Adjusted *P*-value in DESeq2 and FDR in edgeR were used together to determine whether a gene is differentially expressed. If adjusted *P*-value ≤ 0.05 of one gene under DESeq2 and FDR ≤ 0.05 under edgeR, the gene was considered as differentially expressed in between two groups of samples. The intersections of differential expression genes from these two methods were taken as the final results of each comparison. Because we only identified a very small number of the differential expression genes in LOMPE, we merge the results of DESeq2 and edgeR as the final results in the comparison of LOMPE samples with normal samples. The numbers of differentially expressed genes identified by the two methods and gene biotypes were showed in Figure S2A and Table S3.

### The DEGs overlapped with PE-associated genes from the literature

There were a lot of studies to investigate differentially expressed genes compared normal placenta tissues with PE placenta tissues. Due to small sample sizes and undistinguished subtypes of PE samples in previous transcriptomic studies, the differentially expressed genes in PE have not been well defined. To increase the sample sizes, several meta-analyses on reported microarray datasets were published since 2012 84-89. In 2015, Kaartokallio et al reported the first RNA-seq study on PE 50. To obtain a consensus PE-associated genes, we collected 1,177 genes from literature, including 6 meta-analysis papers 84-89, 1 literature-curation paper 90 and 1 RNA-seq research paper 50. These 1,177 genes are PE-associated genes and information for genes were listed in Table S4.

### Pathway enrichment analysis for DEGs of EOSPE-only, LOSPE-only and shared genes

The numbers of differentially expressed protein-coding genes are 2,061 in EOSPE-only, 85 in LOPSE-only and 237 in common (Venn diagram in Figure S3A). KEGG pathway enrichment analysis was done by clusterProfiler package 80. The cutoff of *q*-value ≤ 0.2 was chosen to select most significantly enriched pathways (Figure S3A). There 39 enriched pathways for EOSPE-only DEGs, 35 for LOSPE-only DEGs, and 7 for shared DEGs.

The pathway enrichment for up-regulated and down-regulated DEGs in both EOSPE and LOPSE were done using the same method (Figure S3B-C).

### GO terms enrichment analysis for DEGs of EOSPE and LOSPE

Gene Ontology terms, (BP, CC and MF) enrichment analyses were also using clusterProfiler package 80, and the *q*-value ≤ 0.05 and adjusted *P*-value ≤ 0.05 were set as cutoff to select most significantly enriched terms. The DEGs of EOSPE were statistically significant enriched in 248 terms of biological process (BP), 51 cellular component (CC) and 16 molecular function (MF). The DEGs of LOSPE were statistically significant enriched 169 terms of BP, 22 of CC and 5 of MF. The overlap of three categories of enriched GO terms are 63 (BP), 4 (CC) and 0 (MF) between DEGs of EOSPE and LOSPE, and most of enriched terms are different in EOSPE and LOSPE (Figure S3D-E, and Table S5).

We used semantic similarity-based method (REVIGO) 91 to summarize the enriched GO terms in the tree components. For EOSPE, 199 of 248 BP terms were summarized into 14 representative entries. The first representative entry is the nuclear-transcribed mRNA catabolism, containing 55 terms. The second is SRP-dependent co-translational protein targeting to membrane, containing 42 terms. Other entries include stress-activated protein kinase signaling cascade, regulation of macro-autophagy, response to metal ion, estrogen and insulin, and superoxide metabolism. For LOSPE, however, 130 of 169 BP terms were summarized into 11 representative entries. The first entry is positive regulation of cell adhesion, containing 57 terms, and the second entry was response to interferon-gamma, containing 27 terms. Other entries include response to oxygen levels, temperature homeostasis, epithelial cell proliferation, and synapse organization.

The Enriched CC and MF terms in EOSPE and LOSPE also showed considerable difference (Table S5). This suggested that besides 'canonical pathways' such as HIF-1 signaling pathway, the enriched terms are associated with metabolisms, substrates transport in EOSPE, whereas enriched terms are associated with immune functions in LOSPE.

We found that there are 5 terms of MF enriched in EOSPE DEGs are related to glucose/carbohydrate transport, and 20 terms of BP enriched in EOSPE DEGs are also associated with materials transmembrane transporters, while 17 terms of BP enriched in LOSPE DEGs are associated with transporters.

### Clustering analysis for GO-term-to-gene-bipartite networks for differentially expressed transporter genes of EOSPE and LOSPE

We found that transporter genes were significantly enriched in the DEGs of the EOSPE (*P* = 0.027) compared with all annotated human genes (19,738) in GO, but not significantly enriched DEGs of the LOSPE (*P* = 0.723). There were 205 and 23 transporter genes involved in 397 and 93 GO biological process (GO BP) terms in EOSPE and LOSPE respectively. To summarize the enriched BP terms, we firstly constructed GO-term-to-gene-bipartite networks for EOSPE and LOSPE in Cytoscape using yfiles organic layout. Then we calculated Jaccard score using number of shared genes in the terms, and clustered terms into several clusters based on shared genes. Combining the layout of networks and the results of clustering analysis, 5 and 2 groups of GO BP terms were manually determined for EOSPE and LOSPE, respectively. All enriched BP terms in differentially expressed transporter genes of the EOSPE and the LOSPE were listed in Table S7.

### Transcription factors and target genes

HOMER (Hypergeometric Optimization of Motif EnRichment) 92 was used to search for transcription factor biding sites in DEGs of EOSPE or LOSPE (Table S8).

### Schizophrenia associated genes enriched in the DEGs of the EOSPE and LOSPE

Abnormal intra-uterine environment is an important status of preeclampsia, and our data showed that 90% and 58% babies born by EOSPE and LOSPE patients had FGR/LBW. Recently, a meta-analysis reported that hypertensive disorders of pregnancy may be associated with an increase in the risk of ASD (autism spectrum disorder) and ADHD (attention-deficit/hyperactivity disorder) 71. Weinberger's study showed that the set of genes within genomic risk loci for schizophrenia that have interaction with intra-uterine and perinatal complications is highly expressed in placenta 23. Other studies also demonstrated that preeclampsia was associated with increased risk of offspring schizophrenia 93.

To investigate the relationship between preeclampsia and schizophrenia, we collected 3,437 schizophrenia-associated genes from classical schizophrenia databases and literature 94-100. Since increasing evidences demonstrate the association of preeclampsia with schizophrenia, we want to know if the differentially expressed genes are enriched for schizophrenia associated genes. In our dataset, the protein coding DEGs (2,405, including DEGs from EOSPE, LOSPE and LOMPE), DEGs of EOSPE (2,298) or LOSPE (322) were significant enriched for schizophrenia-associated genes: 20.7% (497/2,405, *P* = 5.0e-5), 20.54% (472/2,298, *P* = 1.4e-4), 22.98% (74/322, *P* = 0.01), compared with the expected 17.8% (the ratio 3,437 schizophrenia associated genes to 19,351 annotated human coding genes). In protein coding DEGs of LOMPE, there is no significant enrichment of schizophrenia associated genes (15.4%, *P* > 0.5) (Figure S2D).

## Supplementary Material

Supplementary figure and table legends.

Supplementary figures.

Supplementary table 1.

Supplementary table 2.

Supplementary table 3.

Supplementary table 4.

Supplementary table 5.

Supplementary table 6.

Supplementary table 7.

Supplementary table 8.

Supplementary table 9.

## Abbreviations

ADHD: attention-deficit/hyperactivity disorder
ASD: autism spectrum disorder
BP: biological process
CC: cellular component
CRP: C-reactive protein
DEGs: differentially expressed genes
EOSPE: early-onset severe PE
FGR: fetal growth restriction
GO: Gene Ontology
GTF: General Transfer Format
HBM: Hemoglobin Subunit Mu
KEGG: Kyoto Encyclopedia of Genes and Genomes
LBW: low birth weight
LOMPE: late-onset mild PE
LOSPE: late-onset severe PE
MF: molecular function
PCA: principal component analysis
PE: Preeclampsia
TFs: transcription factors
