# Supplementary material for: Distinct placental molecular processes associated with early-onset and late-onset preeclampsia
Source: Theranostics. 2021 Mar 5;11(10):5028–44. doi: 10.7150/thno.56141 (PMC7978310; doi:10.7150/thno.56141)
Supplement: Supplementary file 1 — Supplementary figure and table legends. [file thnov11p5028s1.pdf]

Supplementary Information

Distinct placental molecular processes associated with early-onset and late-onset preeclampsia

Zhonglu Ren[1,2,3,4,5,#], Yunfei Gao[1,#], Yue Gao[1,2,3], Guanmei Liang[1,2,3], Qian Chen[1], Sijia Jiang[1], Xiaoxue Yang[1], Cuixia Fan[1], Haizhen Wang[1], Jing Wang[1], Yi-Wu Shi[6], Chaoqun Xiao[1], Mei Zhong[1] and Xinping Yang[1,2,3,7*]

[1] Center for genetics and Developmental Systems Biology, Department of Obstetrics and Gynecology, Nanfang Hospital, Southern Medical University, Guangzhou, 510515, China

[2] Key Laboratory of Mental Health of the Ministry of Education, Guangdong-Hong Kong-Macao Greater Bay Area Center for Brain Science and Brain-Inspired Intelligence and Guangdong Key Laboratory of Psychiatric Disorders, School of Basic Medical Sciences, Southern Medical University, Guangzhou 510515, China

[3] Department of Bioinformatics, School of Basic Medical Sciences, Southern Medical University, Guangzhou, 510515, China

[4] College of Medical Information Engineering, Guangdong Pharmaceutical University, Guangzhou, 510006, China

[5] Medicinal Information & Real-World Engineering Technology Center of Universities in Guangdong Province, Guangzhou, 510006, China

[6] Institute of Neuroscience and Department of Neurology, The Second Affiliated Hospital of Guangzhou Medical University, Guangzhou, 510260, China

[7] Lead Contact

# These authors contribute equally to this work

* To whom correspondence should be addressed: xpyang1@smu.edu.cn, ORCID: 0000-0003-4086-4180

Contents

Supplementary Figures

Supplementary Figure S1. C-reactive protein levels in clinical subtypes and sample clustering analysis based on RNA-seq data of early-onset severe, late-onset severe and mild samples.
Supplementary Figure S2. Identification and validation of differentially expressed genes, and long-term consequence for the offspring.
Supplementary Figure S3. Functional enrichment analysis of differentially expressed genes.
Supplementary Figure S4. Gene-pathway bipartite network of DEGs and KEGG pathways in EOSPE.
Supplementary Figure S5. TFs and Target genes for LOSPE.
Supplementary Figure S6. 'TF-Target-Pathway bipartite networks' for EOSPE.

Supplementary Tables

Supplementary Table S1. Clinical Characteristics.
Supplementary Table S2. Raw counts of RNA-seq data.
Supplementary Table S3. Differentially expressed genes (DEGs).
Supplementary Table S4. PE-associated genes curated from literature.
Supplementary Table S5. Enriched Pathways and GO terms.
Supplementary Table S6. Transporter genes.
Supplementary Table S7. Enriched GO terms and pathways for transporter genes in DEGs of EOSPE and the LOSPE.
Supplementary Table S8. Enriched TF-Target Network.
Supplementary Table S9. Tools, databases and primer sequences.

Supplementary Figure S1-S6

Figure S1. C-reactive protein levels in clinical subtypes and sample clustering analysis based on RNA-seq data of early-onset severe, late-onset severe and mild samples

(A) Box-plot of c-reactive protein levels in LOMPE, EOSPE and LOSPE. The average of normal c-reactive protein levels is 0-5mg/L. **(B)** Heatmap of sample-sample distance for disease samples (late-onset severe, mild and early-onset severe samples). The clustering was performed using Ward.D. Two clear subclasses were observed, where most of late-onset severe together with mild samples clustered as subclass-1 and early-onset severe together with four late-onset severe samples clustered as subclass-2. Top two rows of squares represent the clinical features of the samples: the first row: blue represents fetal growth restriction (FGR) or low birth weight (LBW); gray represents no FGR/LBR; second row: purple represent early-onset (severe) PE; pink late-onset (severe) PE; light green mild PE.

Figure S2. Identification and validation of differentially expressed genes, and long-term consequence for the offspring.

(A) Venn diagrams for differentially expressed genes identified using DESeq2 and edgeR methods in four comparisons. Left Venn diagrams are numbers of up-regulated genes detected by DESeq2 and edgeR and right Venn diagrams are number of down-regulated genes detected by DESeq2 and edgeR. The overlapped genes were taken as the final differentially expressed genes in each comparison group. Light brown represents method of DESeq2; dark brown method of edgeR. **(B)** Box-plots for four up-regulated genes in RNA-seq and q-PCRs. **(C)** Box-plots for six down-regulated genes in RNA-seq and q-PCRs. **(D)** Schizophrenia-associated genes were enriched in DEGs of preeclampsia. The schizophrenia associated genes collected from classical schizophrenia databases and literature showed significantly enrichment in DEGs in all PE samples combined, EOPSE or LOPSE, whereas no enrichment in DEGs in LOMPE. Gray bar represents the expected fraction (17.8%) of schizophrenia associated genes in all protein-coding genes (19,351), the fraction of schizophrenia-associated genes in DEGs in all PE samples (20.7%), the DEGs of EOSPE

(20.54%), LOSPE (22.98%) and LOMPE (15.4%) were presented as red, purple, pink and light green bars. The *P*-value was calculated using the Fisher's exact test, and error bars represent the standard error of the fraction, estimated using bootstrapping with 100 resamplings.

Figure S3. Functional enrichment analysis of differentially expressed genes.

(A) The enriched KEGG pathways with DEGs of EOSPE, LOSPE or intersection between EOSPE and LOSPE. Venn-diagram shows the overlaps between DEGs of EOSPE and LOSPE. **(B-C)** The enriched KEGG pathways with up- and down-regulated DEGs of EOSPE (B) and LOPSE (C). To save figure space, "*" is used to label the shortened terms, and the complete terms are: * (1) Intestinal immune network for IgA production (2) AGE−RAGE signaling pathway in diabetic complications in (C). **(D)** The enriched GO-BP terms for DEGs of EOSPE and LOSPE. To save figure space, "*" is used to label the shortened terms, and the complete terms are: * (1) Nuclear−transcribed mRNA catabolic process, nonsense−mediated decay (2) SRP−dependent cotranslational protein targeting to membrane (3) Positive regulation of protein serine/threonine kinase activity. **(E)** The enriched GO-CC terms for DEGs of EOSPE and LOSPE. Dot colors indicate enrichment *P*-values and dot sizes represent gene ratios in the enriched pathways. To save figure space, "*" is used to label the shortened terms, and the complete terms are: * (1) Integral component of lumenal side of endoplasmic reticulum membrane (2) Lumenal side of endoplasmic reticulum membrane.

Figure S4. Gene-pathway bipartite network of DEGs and KEGG pathways in EOSPE.

Red nodes represent up-regulated genes and green nodes are down-regulated genes in EOSPE; and yellow diamond nodes represent names of the enriched KEGG pathways. The edges connect the DEGs to the KEGG pathways the genes belong to.

Figure S5. TFs and Target genes for LOSPE.

(A) Transcription factor-binding motifs were searched in the DEGs of LOSPE. Of 375 DEGs

in LOSPE, 80% (301/375) were predicted to be targeted by 9 TFs. **(B)** TF-targets network for the DEGs of LOPSE. Of the 9 enriched TFs (the triangles), 6 TFs (pink circles) target the up-regulated DEGs, 3 TFs (blue circles) target the down-regulated DEGs. The up-regulated targets are in the red nodes (246), and the down-regulated targets are in the blue nodes (55). **(C)** The Sankey diagram showing the relationship between TFs and the enriched pathways with its targets. Enrichment analysis was performed on the targets of each TF. A total number of 32 enriched pathways (the right column) were found for 7 TFs (colored column in the left). The binding motifs corresponding to TFs are listed on the left. The orange color in the right bar indicates the pathways that are overlapped with pathways enriched with DEGs of LOSPE (Figure 3B), and the light blue color in the right bar indicates pathways that are newly-found enriched pathways with the targets of TFs. **(D-E)** 'TF-Target-Pathway bipartite sub-networks' for TFs *HOXD11* and *SOX21*. The *HOXD11* targeting DEGs are involved in 19 pathways, which are mostly related to immune and inflammation function **(D)**. The *HIF1A* targeting DEGs are involved in two pathways, one of which is known to be involved in immune and inflammation function **(E).** Circles: non-TF genes; triangles: TFs; yellow diamonds: KEGG pathways; red: up-regulation; blue: down-regulation; light blue: no expression change.

Figure S6. 'TF-Target-Pathway bipartite networks' for EOSPE.

'TF-Target-Pathway bipartite sub-networks' for *E2F1* (A), *HOXD11* (B), *IRF4* (C), *MITF* (D), *PRDM1* (E), *RELA* (F), *TEAD2* (G), *TIGF2* (H). Circles: non-TF genes; triangles: TFs; yellow diamonds: KEGG pathways; red: up-regulation; blue: down-regulation; light blue: no expression change.

Supplementary Table S1-S9

Tables are provided in excel files.

Table S1. Clinical characteristics.

Sheet1: Clinical characteristics comparisons among groups of patients with EOSPE, LOSPE or LOMPE and normal subjects;

Sheet2: Clinical characteristics comparison between subclass-1 and subclass-2 PE sample groups.

Table S2. Raw counts of RNA-seq data.

Sheet1: Raw counts of each gene for two cord blood samples;

Sheet2: Raw counts of each gene for 65 placental samples before and after removing blood contamination.

Table S3. Differentially expressed genes (DEGs).

Sheet1: Gene biotypes and direction of difference of DEGs;

Sheet2: DEGs of All PE;

Sheet3: DEGs of EOSPE;

Sheet4: DEGs of LOSPE;

Sheet5: DEGs of LOMPE;

Sheet6: Q-PCR validation.

Table S4. PE-associated genes curated from literature.

Sheet1: Literature information, number of PE-associated genes and datasets used in these studies;

Sheet2: PE-associated genes with times found in the literature and the overlap with DEGs of EOSPE, LOPSE and LOMPE.

Table S5. Enriched Pathways and GO terms.

Sheet1: Enriched KEGG pathways in the DEGs of the EOSPE and LOSPE;

Sheet2: Enriched KEGG pathways in the DEGs of the EOSPE-only, LOSPE-only and shared genes in Sheet1;

Sheet3: Enriched KEGG pathways in the up- and down-regulated DEGs of the EOSPE and up-regulated DEGs of the LOSPE;

Sheet4: Enriched GO BP terms;

Sheet5: Enriched GO CC terms;

Sheet6: Enriched GO MF terms;

Sheet7: Representative term groups in BP, CC and MF.

Table S6. Transporter genes.

Sheet1: Information for 1,554 transporter genes downloaded from GO and overlapped genes in the DEGs of the EOSPE and the LOSPE;

Sheet2: Information for differentially expressed transporter genes in the EOSPE and the LOSPE.

Table S7. Enriched GO terms and pathways for transporter genes in DEGs of EOSPE and the LOSPE.

Sheet1: Information in the GO-term-to-gene-bipartite networks of EOPSE and LOPSE;

Sheet2: Enriched 397 BP terms table in EOSPE;

Sheet3: Enriched 93 BP terms table in LOPSE;

Sheet4: Enriched KEGG pathways in differentially expressed transporter genes of the EOSPE.

Table S8. Enriched TF-Target Network.

Sheet1: Information of the enriched TF-binding motifs in the DEGs of EOSPE;

Sheet2: TF-Target network of EOSPE;

Sheet3: The enriched KEGG Pathways with TF targets in EOSPE;

Sheet4: Information of the enriched TF-binding motifs in the DEGs of LOSPE;

Sheet5: TF-Target network of LOSPE;

Sheet6: The enriched KEGG Pathways with TF targets in LOSPE.

Table S9. Tools, databases and primer sequences.

Sheet1: Tools for analyses;

Sheet2: Databases;

Sheet3: Primer sequences for qPCR.
