# Supplementary material for: Distinct placental molecular processes associated with early-onset and late-onset preeclampsia
Source: Theranostics. 2021 Mar 5;11(10):5028–44. doi: 10.7150/thno.56141 (PMC7978310; doi:10.7150/thno.56141)
Supplement: Supplementary file 2 — Supplementary figures. [file thnov11p5028s2.pdf]

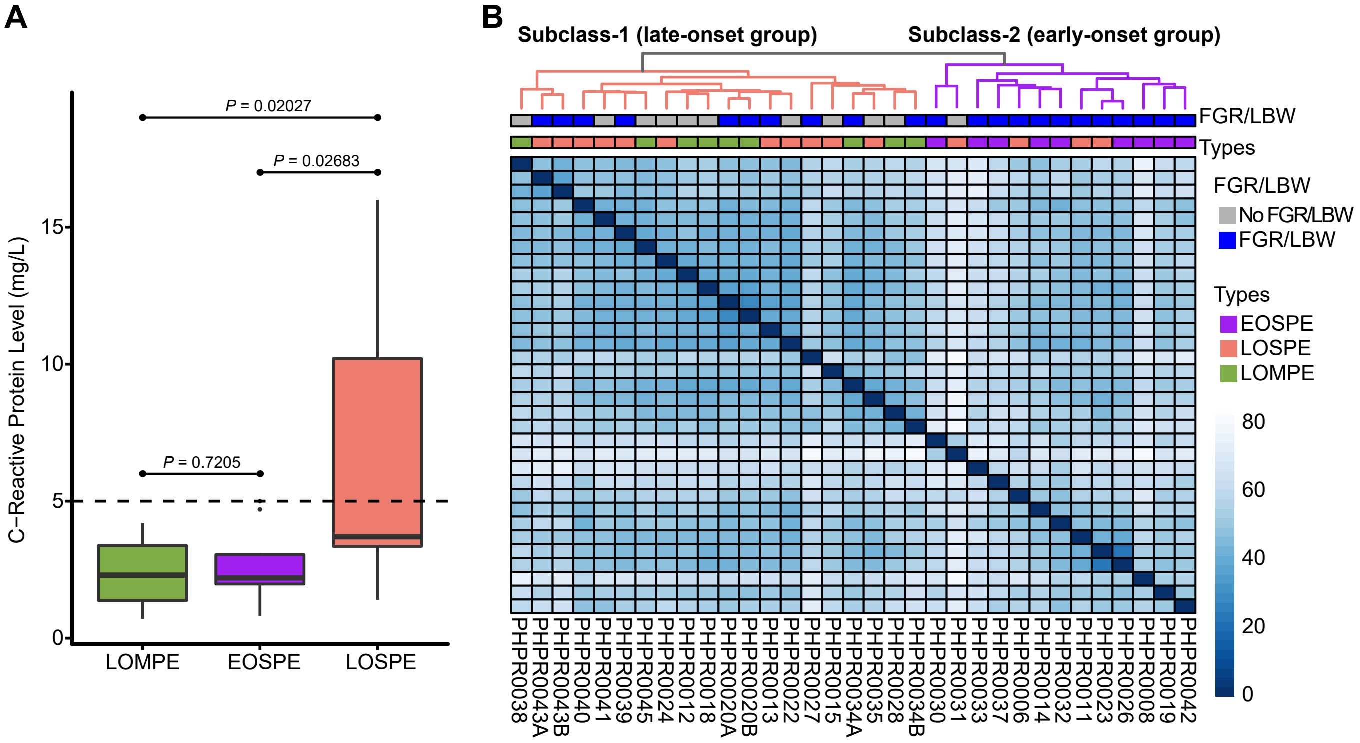

Figure S1. C-reactive protein levels in clinical subtypes and sample clustering analysis based on RNA-seq data of early-onset severe, late-onset severe and mild samples.

(A) Box-plot of c-reactive protein levels in LOMPE, EOSPE and LOSPE. The average of normal c-reactive protein levels is 0-5mg/L. **(B)** Heatmap of sample-sample distance for disease samples (late-onset severe, mild and early-onset severe samples). The clustering was performed using Ward.D. Two clear subclasses were observed, where most of late-onset severe together with mild samples clustered as subclass-1 and early-onset severe together with four late-onset severe samples clustered as subclass-2. Top two rows of squares represent the clinical features of the samples: the first row: blue represents fetal growth restriction (FGR) or low birth weight (LBW); gray represents no FGR/LBR; second row: purple represent early-onset (severe) PE; pink late-onset (severe) PE; light green mild PE.

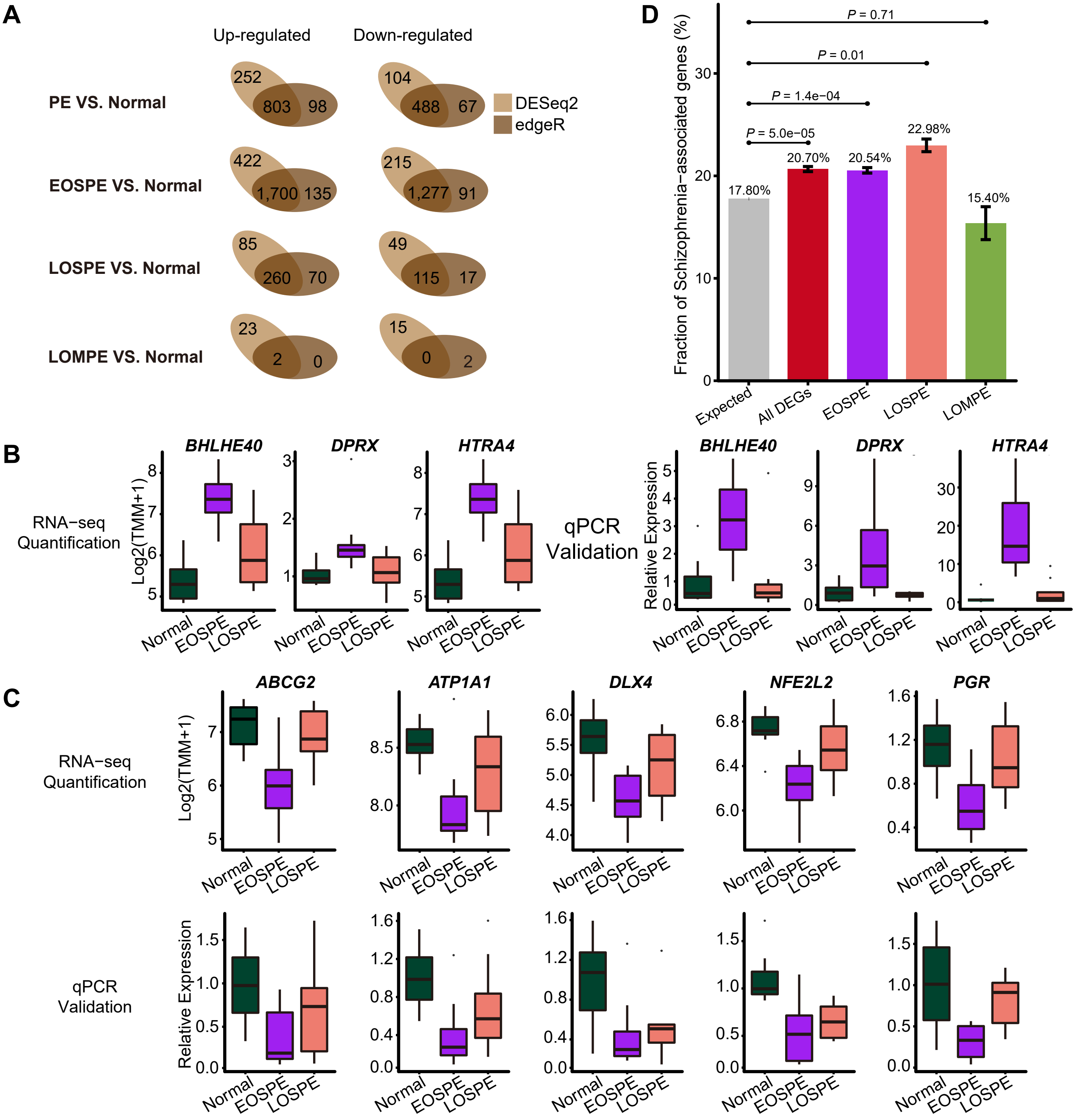

Figure S2. Identification and validation of differentially expressed genes, and long-term consequence for the offspring.
(A) Venn diagrams for differentially expressed genes identified using DESeq2 and edgeR methods in four comparisons. Left Venn diagrams are numbers of up-regulated genes detected by DESeq2 and edgeR and right Venn diagrams are number of down-regulated genes detected by DESeq2 and edgeR. The overlapped genes were taken as the final differentially expressed genes in each comparison group. Light brown represents method of DESeq2; dark brown method of edgeR. **(B)** Box-plots for four up-regulated genes in RNA-seq and q-PCRs. **(C)** Box-plots for six down-regulated genes in RNA-seq and q-PCRs. **(D)** Schizophrenia-associated genes were enriched in DEGs of preeclampsia. The schizophrenia associated genes collected from classical schizophrenia databases and literature showed significantly enrichment in DEGs in all PE samples combined, EOPSE or LOPSE, whereas no enrichment in DEGs in LOMPE. Gray bar represents the expected fraction (17.8%) of schizophrenia associated genes in all protein-coding genes (19,351), the fraction of schizophrenia-associated genes in DEGs in all PE samples (20.7%), the DEGs of EOSPE (20.54%), LOSPE (22.98%) and LOMPE (15.4%) were presented as red, purple, pink and light green bars. The *P*-value was calculated using the Fisher's exact test, and error bars represent the standard error of the fraction, estimated using bootstrapping with 100 resamplings.

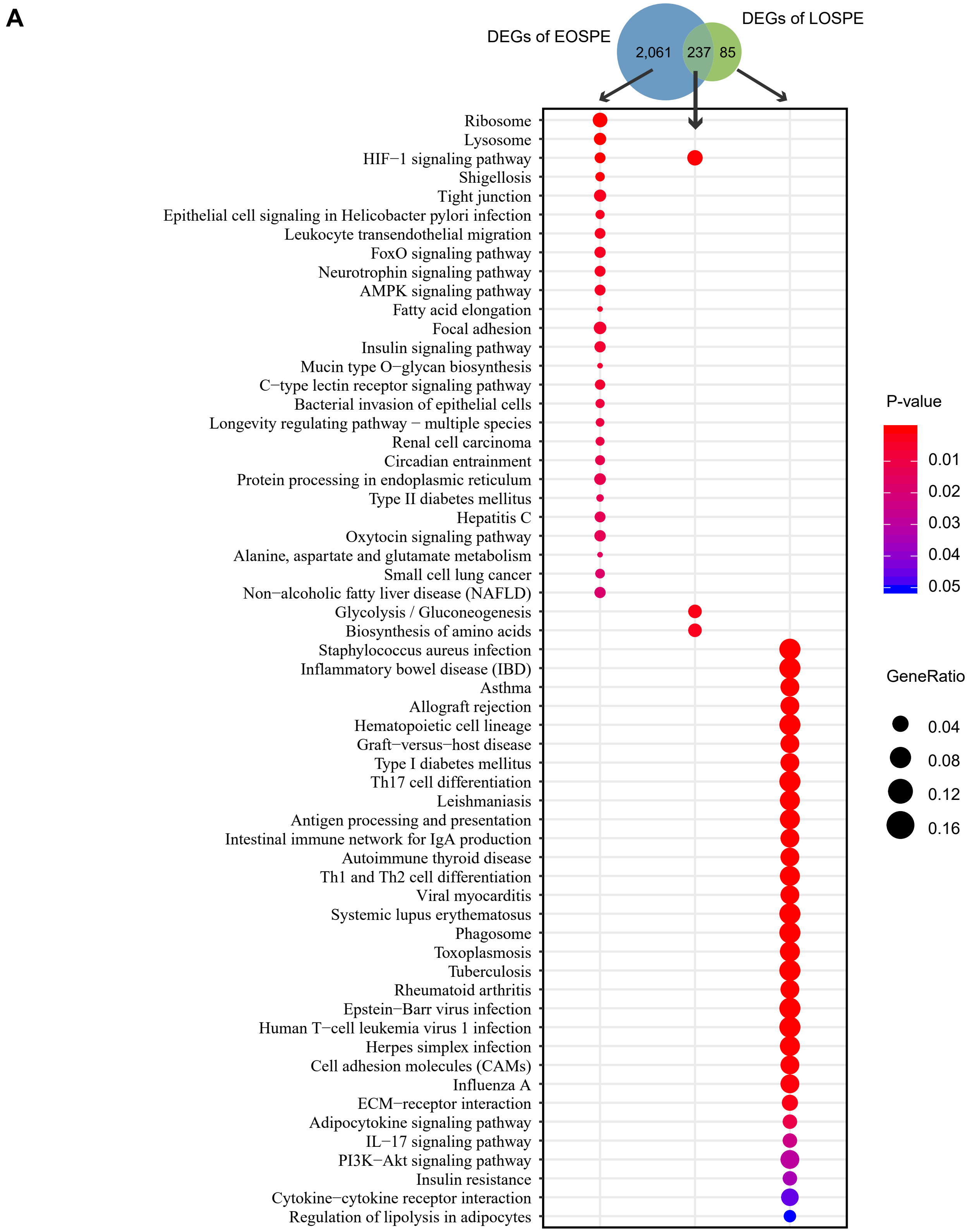

Figure S3. Functional enrichment analysis of differentially expressed genes.
(A) The enriched KEGG pathways with DEGs of EOSPE, LOSPE or intersection between EOSPE and LOSPE. Venn-diagram shows the overlaps between DEGs of EOSPE and LOSPE. **(B-C)** The enriched KEGG pathways with up- and down-regulated DEGs of EOSPE **(B)** and LOPSE **(C)**. To save figure space, "*" is used to label the shortened terms, and the complete terms are: * (1) Intestinal immune network for IgA production (2) AGE−RAGE signaling pathway in diabetic complications in **(C)**. **(D)** The enriched GO-BP terms for DEGs of EOSPE and LOSPE. To save figure space, "*" is used to label the shortened terms, and the complete terms are: * (1) Nuclear−transcribed mRNA catabolic process, nonsense−mediated decay (2) SRP−dependent cotranslational protein targeting to membrane (3) Positive regulation of protein serine/threonine kinase activity. **(E)** The enriched GO-CC terms for DEGs of EOSPE and LOSPE. Dot colors indicate enrichment *P*-values and dot sizes represent gene ratios in the enriched pathways. To save figure space, "*" is used to label the shortened terms, and the complete terms are: * (1) Integral component of lumenal side of endoplasmic reticulum membrane (2) Lumenal side of endoplasmic reticulum membrane.

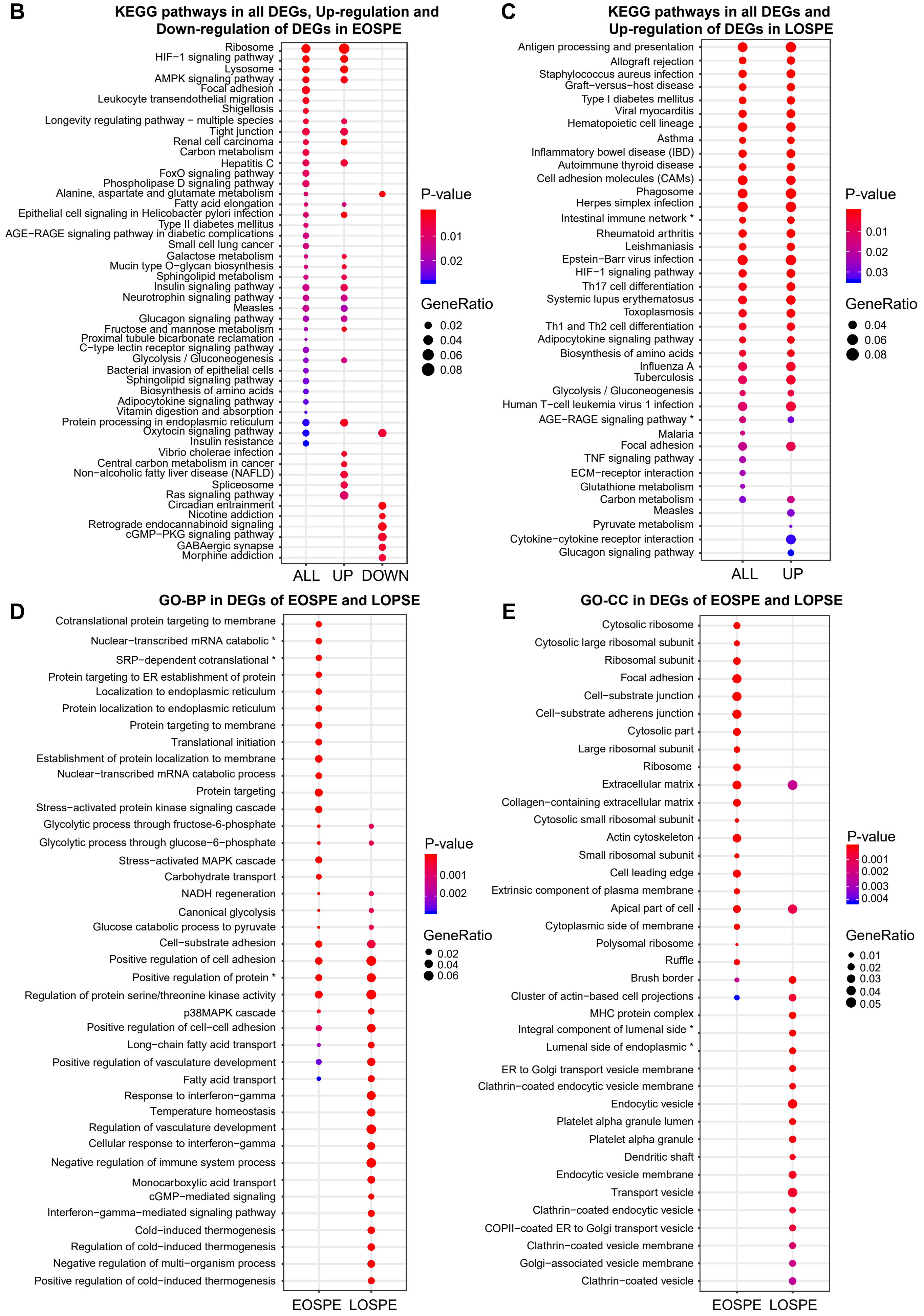
B
KEGG pathways in all DEGs, Up-regulation and Down-regulation of DEGs in EOSPE
Ribosome
HIF−1 signaling pathway
Lysosome
AMPK signaling pathway
Focal adhesion
Leukocyte transendothelial migration
Shigellosis
Longevity regulating pathway − multiple species
Tight junction
Renal cell carcinoma
Carbon metabolism
Hepatitis C
FoxO signaling pathway
Phospholipase D signaling pathway
Alanine, aspartate and glutamate metabolism
Fatty acid elongation
Epithelial cell signaling in Helicobacter pylori infection
Type II diabetes mellitus
AGE−RAGE signaling pathway in diabetic complications
Small cell lung cancer
Galactose metabolism
Mucin type O−glycan biosynthesis
Sphingolipid metabolism
Insulin signaling pathway
Neurotrophin signaling pathway
Measles
Glucagon signaling pathway
Fructose and mannose metabolism
Proximal tubule bicarbonate reclamation
C−type lectin receptor signaling pathway
Glycolysis / Gluconeogenesis
Bacterial invasion of epithelial cells
Sphingolipid signaling pathway
Biosynthesis of amino acids
Adipocytokine signaling pathway
Vitamin digestion and absorption
Protein processing in endoplasmic reticulum
Oxytocin signaling pathway
Insulin resistance
Vibrio cholerae infection
Central carbon metabolism in cancer
Non−alcoholic fatty liver disease (NAFLD)
Spliceosome
Ras signaling pathway
Circadian entrainment
Nicotine addiction
Retrograde endocannabinoid signaling
cGMP−PKG signaling pathway
GABAergic synapse
Morphine addiction
ALL
UP
DOWN
P-value
0.01
0.02
GeneRatio
0.02
0.04
0.06
0.08
C
KEGG pathways in all DEGs and Up-regulation of DEGs in LOSPE
Antigen processing and presentation
Allograft rejection
Staphylococcus aureus infection
Graft−versus−host disease
Type I diabetes mellitus
Viral myocarditis
Hematopoietic cell lineage
Asthma
Inflammatory bowel disease (IBD)
Autoimmune thyroid disease
Cell adhesion molecules (CAMs)
Phagosome
Herpes simplex infection
Intestinal immune network *
Rheumatoid arthritis
Leishmaniasis
Epstein−Barr virus infection
HIF−1 signaling pathway
Th17 cell differentiation
Systemic lupus erythematosus
Toxoplasmosis
Th1 and Th2 cell differentiation
Adipocytokine signaling pathway
Biosynthesis of amino acids
Influenza A
Tuberculosis
Glycolysis / Gluconeogenesis
Human T−cell leukemia virus 1 infection
AGE−RAGE signaling pathway *
Malaria
Focal adhesion
TNF signaling pathway
ECM−receptor interaction
Glutathione metabolism
Carbon metabolism
Measles
Pyruvate metabolism
Cytokine−cytokine receptor interaction
Glucagon signaling pathway
ALL
UP
P-value
0.01
0.02
0.03
GeneRatio
0.04
0.06
0.08
D
GO-BP in DEGs of EOSPE and LOSPE
Cotranslational protein targeting to membrane
Nuclear−transcribed mRNA catabolic *
SRP−dependent cotranslational *
Protein targeting to ER establishment of protein
Localization to endoplasmic reticulum
Protein localization to endoplasmic reticulum
Protein targeting to membrane
Translational initiation
Establishment of protein localization to membrane
Nuclear−transcribed mRNA catabolic process
Protein targeting
Stress−activated protein kinase signaling cascade
Glycolytic process through fructose-6-phosphate
Glycolytic process through glucose−6−phosphate
Stress−activated MAPK cascade
Carbohydrate transport
NADH regeneration
Canonical glycolysis
Glucose catabolic process to pyruvate
Cell−substrate adhesion
Positive regulation of cell adhesion
Positive regulation of protein *
Regulation of protein serine/threonine kinase activity
p38MAPK cascade
Positive regulation of cell−cell adhesion
Long−chain fatty acid transport
Positive regulation of vasculature development
Fatty acid transport
Response to interferon−gamma
Temperature homeostasis
Regulation of vasculature development
Cellular response to interferon−gamma
Negative regulation of immune system process
Monocarboxylic acid transport
cGMP−mediated signaling
Interferon−gamma−mediated signaling pathway
Cold−induced thermogenesis
Regulation of cold−induced thermogenesis
Negative regulation of multi−organism process
Positive regulation of cold−induced thermogenesis
EOSPE
LOSPE
P-value
0.001
0.002
GeneRatio
0.02
0.04
0.06
E
GO-CC in DEGs of EOSPE and LOSPE
Cytosolic ribosome
Cytosolic large ribosomal subunit
Ribosomal subunit
Focal adhesion
Cell−substrate junction
Cell−substrate adherens junction
Cytosolic part
Large ribosomal subunit
Ribosome
Extracellular matrix
Collagen−containing extracellular matrix
Cytosolic small ribosomal subunit
Actin cytoskeleton
Small ribosomal subunit
Cell leading edge
Extrinsic component of plasma membrane
Apical part of cell
Cytoplasmic side of membrane
Polysomal ribosome
Ruffle
Brush border
Cluster of actin−based cell projections
MHC protein complex
Integral component of lumenal side *
Lumenal side of endoplasmic *
ER to Golgi transport vesicle membrane
Clathrin−coated endocytic vesicle membrane
Endocytic vesicle
Platelet alpha granule lumen
Platelet alpha granule
Dendritic shaft
Endocytic vesicle membrane
Transport vesicle
Clathrin−coated endocytic vesicle
COPII−coated ER to Golgi transport vesicle
Clathrin−coated vesicle membrane
Golgi−associated vesicle membrane
Clathrin−coated vesicle
EOSPE
LOSPE
P-value
0.001
0.002
0.003
0.004
GeneRatio
0.01
0.02
0.03
0.04
0.05

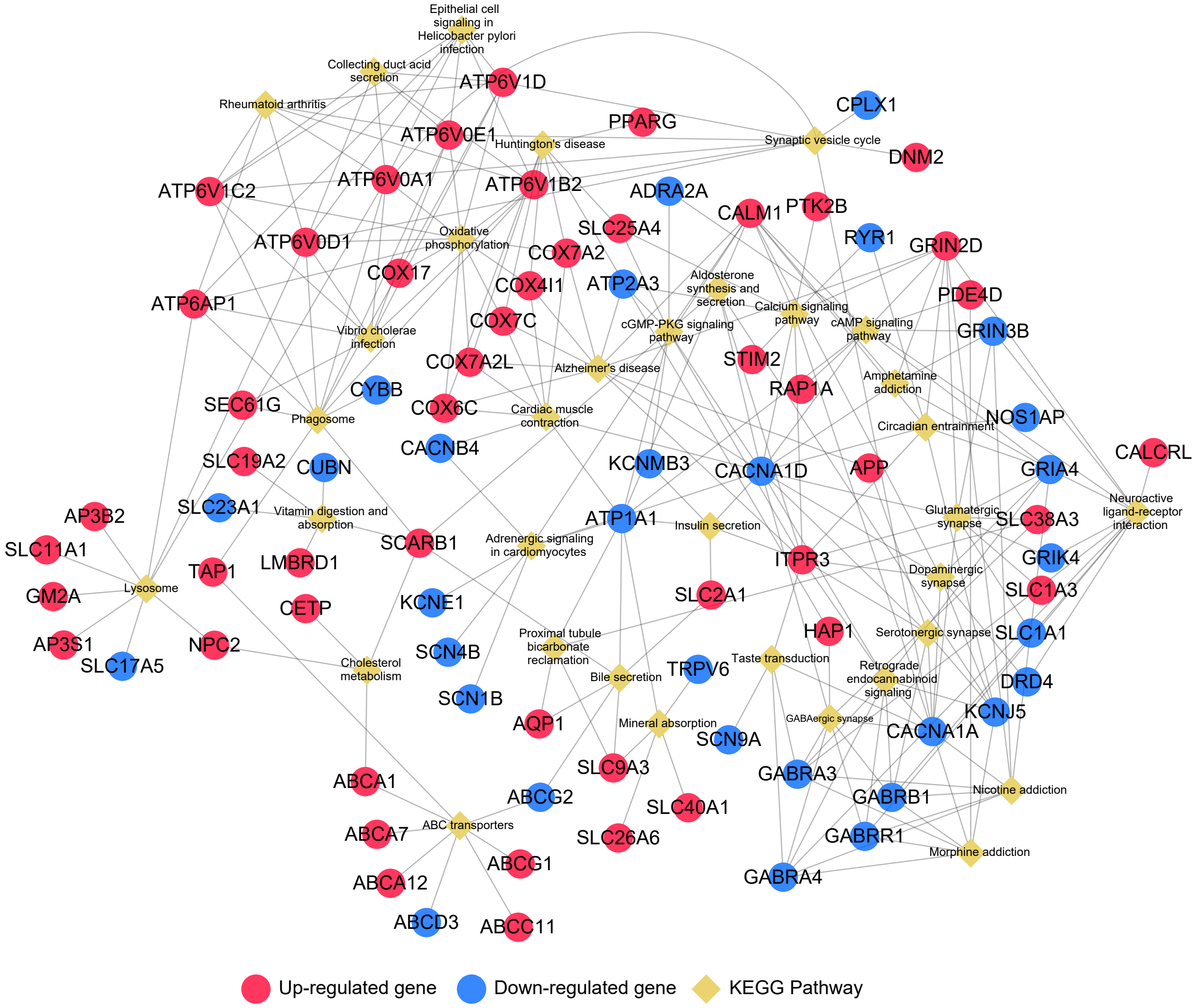

Figure S4. Gene-pathway bipartite network of DEGs and KEGG pathways in EOSPE.
Red nodes represent up-regulated genes and green nodes are down-regulated genes in EOSPE; and yellow diamond nodes represent names of the enriched KEGG pathways. The edges connect the DEGs to the KEGG pathways the genes belong to.

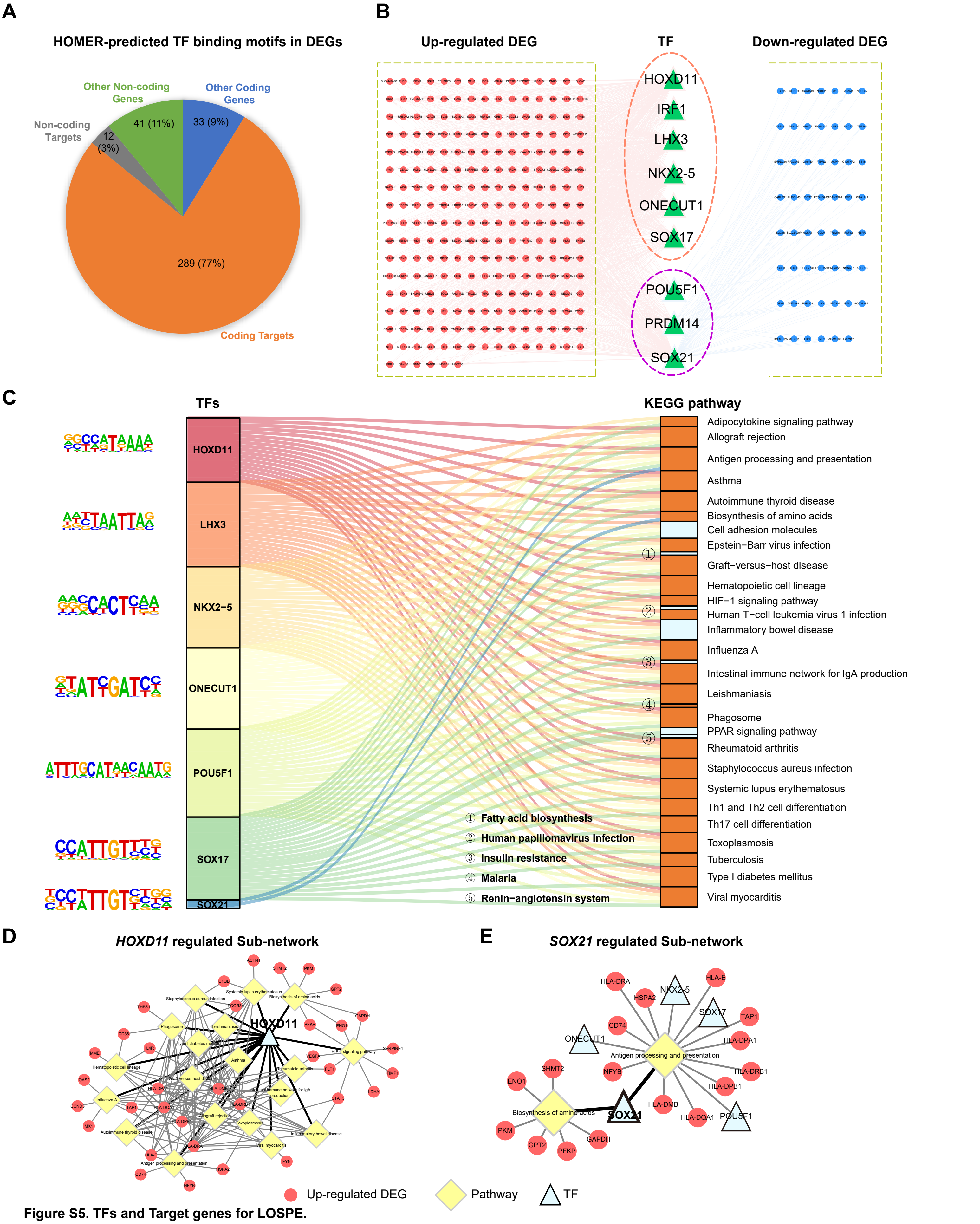

Figure S5. TFs and Target genes for LOSPE.

(A) Transcription factor-binding motifs were searched in the DEGs of LOSPE. Of 375 DEGs in LOSPE, 80% (301/375) were predicted to be targeted by 9 TFs. **(B)** TF-targets network for the DEGs of LOPSE. Of the 9 enriched TFs (the triangles), 6 TFs (pink circles) target the up-regulated DEGs, 3 TFs (blue circles) target the down-regulated DEGs. The up-regulated targets are in the red nodes (246), and the down-regulated targets are in the blue nodes (55). **(C)** The Sankey diagram showing the relationship between TFs and the enriched pathways with its targets. Enrichment analysis was performed on the targets of each TF. A total number of 32 enriched pathways (the right column) were found for 7 TFs (colored column in the left). The binding motifs corresponding to TFs are listed on the left. The orange color in the right bar indicates the pathways that are overlapped with pathways enriched with DEGs of LOSPE (Figure 3B), and the light blue color in the right bar indicates pathways that are newly-found enriched pathways with the targets of TFs. **(D-E)** 'TF-Target-Pathway bipartite sub-networks' for TFs *HOXD11* and *SOX21* . The *HOXD11* targeting DEGs are involved in 19 pathways, which are mostly related to immune and inflammation function **(D)**. The *HIF1A* targeting DEGs are involved in two pathways, one of which is known to be involved in immune and inflammation function **(E)**. Circles: non-TF genes; triangles: TFs; yellow diamonds: KEGG pathways; red: up-regulation; blue: down-regulation; light blue: no expression change.

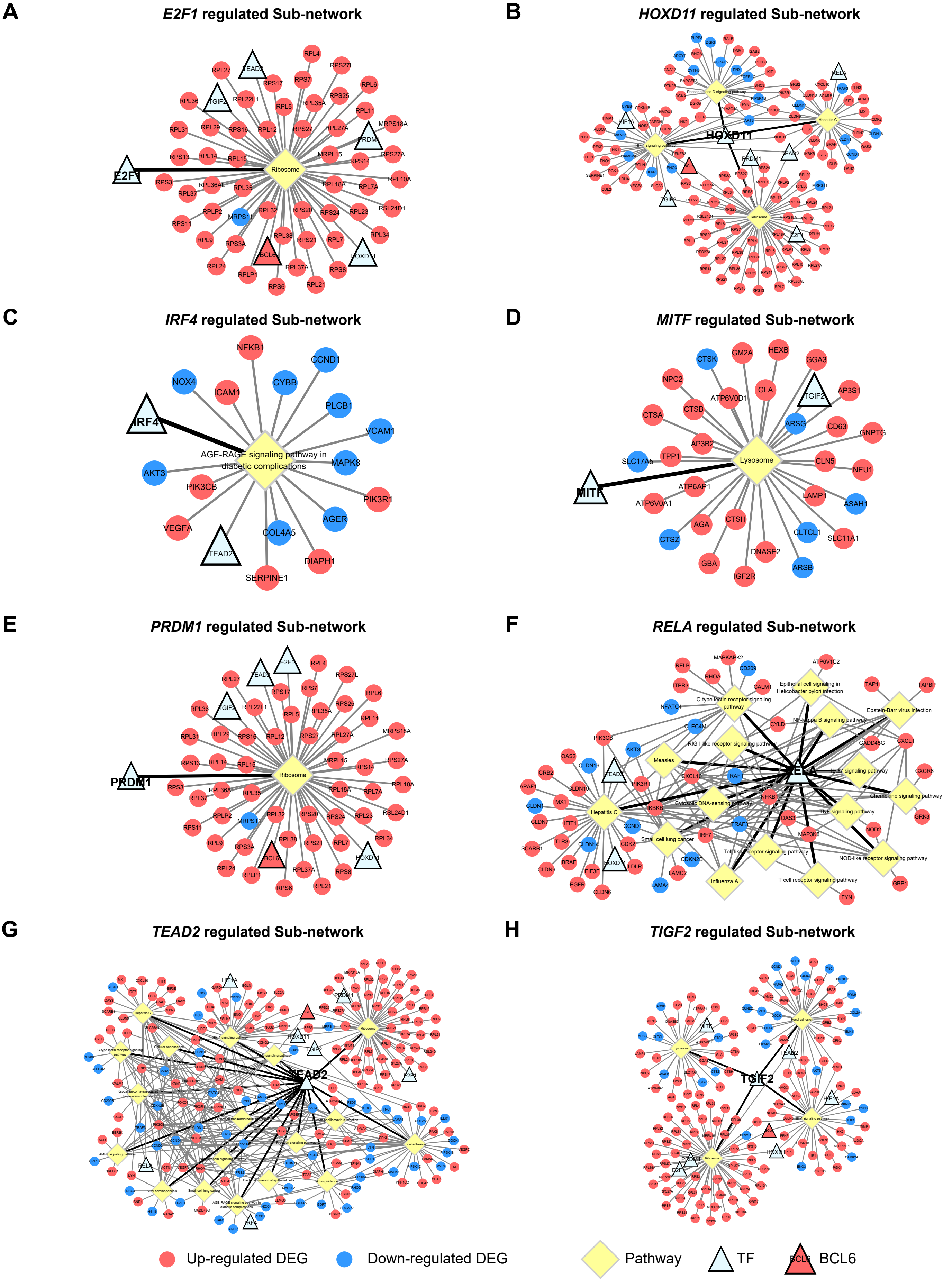

Figure S6. 'TF-Target-Pathway bipartite networks' for EOSPE.
'TF-Target-Pathway bipartite sub-networks' for *E2F1* **(A)**, *HOXD11* **(B)**, *IRF4* **(C)**, *MITF* **(D)**, *PRDM1* **(E)**, *RELA* **(F)**, *TEAD2* **(G)**, *TIGF2* **(H)**. Circles: non-TF genes; triangles: TFs; yellow diamonds: KEGG pathways; red: up-regulation; blue: down-regulation; light blue: no expression change.
